# The Effects of Biochar on the Revival and Performance of an Organohalide-Respiring Mixed Culture

**DOI:** 10.1021/acs.est.5c13638

**Published:** 2026-04-03

**Authors:** Weilun Zhao, Hongyu Dang, Han Cao, Sumbul Hafeez, Wenqing Xu, Timothy E. Mattes

**Affiliations:** † Department of Civil and Environmental Engineering, 4083 The University of Iowa, 4105 Seamans Center, Iowa City, Iowa 52242, United States; ‡ Department of Civil Engineering, 8210 Villanova University, 140 Tolentine Hall, Villanova, Pennsylvania 19085, United States

**Keywords:** bioremediation, SDC-9, *Dehalococcoides*, methanogens, reductive dehalogenase, biochar

## Abstract

Anaerobic reductive dechlorination of chlorinated ethenes (CEs) in groundwater, driven by bioaugmentation of organohalide-respiring bacteria (OHRB), can stall when OHRB abundance and activity are low, leading to incomplete dechlorination and daughter-product accumulation. Pyrogenic carbonaceous matter (PCM), increasingly applied as CE sorbents in groundwater, may enhance OHRB performance. We evaluated how poplar biochars pyrolyzed from 350 to 900 °C influence ethene formation and methanogenesis in an anaerobic tetrachloroethene (PCE)—dechlorinating consortium with initially low OHRB activity. The stressed consortium accumulated *cis*-dichloroethene and produced no ethene in controls without biochar (no materials and sand), but completely dechlorinated PCE to ethene in all biochar treatments. Compared to controls, OHRB in biochar treatments more strongly expressed genes for biofilm formation, resuscitation, cobalamin transport and salvage, and pilus formation, indicating their involvement in OHRB revival in the presence of biochar. Ethene production rates were higher with less apolar biochars produced at 350–500 °C (5.1–5.6 μmol/bottle/day) than with more apolar biochars produced at 700–900 °C (3.2–3.7 μmol/bottle/day). A positive correlation between ethene formation rate and biochar pore size suggests that CE pore-filling and desorption hysteresis affect ethene production. These results identify material properties that can be tuned to enhance targeted biological activity and inform PCM-based CE bioremediation strategies.

## Graphical abstract

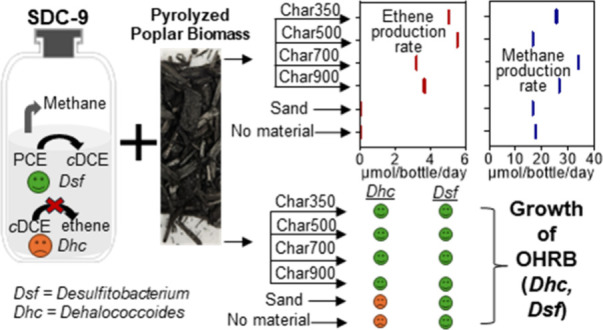

## Introduction

Anaerobic bioremediation is employed to clean up groundwater and sediments polluted by chlorinated ethenes (CEs; e.g., tetrachloroethene (PCE) and trichloroethene (TCE)).^1,2^ Under anaerobic groundwater conditions, PCE and TCE dechlorination by organohalide-respiring bacteria (OHRB) is a stepwise process mediated by reductive dehalogenase enzymes, first generating *cis*-dichloroethene (*cis*-DCE) and then vinyl chloride (VC) intermediates prior to forming the final product ethene.^1,2^ However, *cis*-DCE and VC, which are toxic intermediates of greater concern than the parent CEs, often accumulate in groundwater during anaerobic CE bioremediation.^3–6^

Although OHRB occur naturally in many polluted aquifers, their populations and/or activity are often too low to achieve complete dechlorination of CEs to ethene.^3,7^ Biostimulation and bioaugmentation strategies are therefore used to increase OHRB abundance, enhance OHRB activity, and improve CE dechlorination rates in situ.^3,7–9^ Biostimulation involves introducing electron donors into the subsurface, while bioaugmentation involves adding consortia containing active OHRB such as *Dehalococcoides mccartyi* (*Dhc*).^10,11^ Commercially available mixed PCE-dechlorinating consortia such as SDC-9 and KB-1 are widely used in bioaugmentation applications for CE biodegradation.^9,12–14^ Despite this, field bioaugmentation applications of OHRB can suffer from lag phases and *cis*-DCE or VC accumulation. These limitations arise from factors such as low electron donor availability, competition with other microbes, or unfavorable redox conditions,^15–20^ highlighting the need for approaches that improve OHRB activity under stressed conditions.

Past research suggests that pyrogenic carbonaceous matter (PCM) such as biochar or activated carbons has a beneficial effect on reductive dechlorination of CEs and chlorinated ethanes.^21–24^ This reactivity was mostly attributed to sorption of CEs by PCM, which decreased CE toxicity, thereby creating a more favorable environment for suspended microbes.^22–24^ Oxygen-containing PCM functional groups (e.g., quinone and hydroquinone) and PCM conductivity are thought to facilitate redox reactions by promoting electron transfer.^25^ Micropore size and abundance in PCM influence the sorption and desorption behavior of small molecules such as CEs,^25–28^ while macropores likely provide microbial habitats. However, the role of specific PCM properties on microbial dechlorination processes is largely inferred. More research is needed to understand the impacts of PCM properties (e.g., redox characteristics, surface functionality, and pore structure) on CE dechlorination by OHRB and the supporting microbial community. These knowledge gaps limit efforts to optimize PCM for enhanced CE bioremediation strategies.

The purpose of this study was to investigate the effects of PCM (i.e., biochar) properties on PCE dechlorination performance (i.e., ethene production) of SDC-9 cultures, especially when the initial abundance and activity of the critical OHRB, *Dehalococcoides*, is low. To better reflect practical bioremediation applications in the field, especially in groundwater environments where *Dehalococcoides* populations may be low or dormant, a dormant SDC-9 culture, where PCE dechlorination had stalled at *cis*-DCE, was used to mimic such stressed conditions. *Dehalococcoides* and methanogen population dynamics were quantified by qPCR.^29–31^ Metagenomic and metatranscriptomic sequencing provided OHRB and methanogen gene abundance and expression information. Biochar properties were characterized and correlated with ethene formation rates. By exploring the role of biochar in reactivating dormant PCE-dechlorinating cultures, the study provides insights into how tailoring PCM properties could impact dechlorinating communities in subsurface groundwater systems where environmental conditions often limit microbial activity and thereby enhance CE bioremediation performance.

## Materials and Methods

### SDC-9 Culture, Growth Medium, and Chemicals

An anaerobic PCE-dehalogenating culture^9^ (SDC-9; APTIM, Lawrenceville, NJ) was used in both dormant and active states in this study. The dormant SDC-9 culture had been stored at 4 °C without feeding for approximately 2 years and contained ∼10^6^ *Dhc* cells/mL, whereas the active SDC-9 culture was obtained fresh from APTIM and contained >1 × 10^11^ *Dhc* cells/mL at the time of the experiments. A buffered, reduced anaerobic mineral medium (RAMM),^32^ without vitamins, was used for culturing and experiments to better simulate subsurface conditions, where exogenous vitamins are typically absent and microbial communities depend on endogenous synthesis or transfer.^33,34^ Serum bottles (160 mL; Wheaton, DWK Life Sciences, Novato, CA) containing RAMM (93 mL) were sealed with blue butyl rubber stoppers (20 mm, Chemglass Life Sciences, Vineland, NJ) and aluminum crimp caps (20 mm, Wheaton). RAMM was reduced by sparging with filter-sterilized high-purity 80% N_2_/20% CO_2_ gas (Praxair Inc., Toledo, OH) for at least 20 min. Reducing agents (0.2 mL 200 mM sodium sulfide (Na_2_S·9H_2_O, 98+%, Acros Organics, Fair Lawn, NJ)) and 0.2 mL 200 mM l-cysteine HCl monohydrate (Research Products International Corp., Mt. Prospect, IL)) were added to remove residual oxygen. Reducing agents and sodium lactate solution (60% w/w; Sigma-Aldrich, St. Louis, MO) were filter-sterilized and prepared anaerobically by sparging with N_2_ gas (Linde Inc., Danbury, CT).

PCE (≥99.9%), TCE (≥99.9%), and *cis*-DCE (97%) were obtained from Sigma-Aldrich. VC (99%, Synquest Laboratories, Alachua, FL), ethene (99.5%, Specialty Gases of America, Toledo, OH), and methane (99.0%, Praxair INC, Danbury, CT) were used for gas chromatography (GC) standards and controls. Acetate, propionate, and butyrate (≥99%, Sigma-Aldrich) were used for ion chromatography (IC) standards. Washed SiO_2_ sand from Alfa Aesar (Ward Hill, MA) served as a surface control for comparison with biochar.

### Biochar Preparation and Characterization

Biochar was produced by pyrolysis of poplar (*Liriodendron tulipifera*) biomass at four different temperatures (i.e., 350, 500, 700, and 900 °C) under oxygen-limited conditions (i.e., without active gas flow) for 2 h in a closed muffle furnace (model 550-58, Fisher Scientific, USA), as described previously.^35^ The different pyrolysis temperatures were chosen to provide a range of biochar material properties that could influence sorption and microbial community behavior. Biochar yields were 52.5, 36, 37.5, and 30.0% (by weight). After pyrolysis, biochar was cooled in air, ground with a mortar and pestle, and passed through 250 and 150 μm sieves. Chars in the 150–250 μm size range were used for experiments. All biochar samples used for experiments were derived from the same production batch. Chars are hereafter identified as Char*X*, where *X* represents the pyrolysis temperature.

All four chars were analyzed on a NOVA 3000e (Quantachrome Instruments, Boynton Beach, FL, U.S.A.) using nitrogen adsorption and desorption (Figure S1) from 0.0125 to 0.98 *p*/*p*_0_ at 77.3 K, and with CO_2_ (Figure S2) at 273 K with up to 0.03 *p*/*p*_0_ (Particle Technology Labs, Downers Grove, IL, U.S.A.) to determine the surface area and pore-size distribution (Figure S3). Additional biochar properties, including the ζ potential (Figure S4), X-ray photoelectron spectroscopy (XPS) spectra, elemental analysis, energy dispersive spectroscopy (EDS), conductivity, and electron-donating capacity (EDC) of biochar (Figure S5), were determined as described in Section S1.

### Experimental Setup

The SDC-9 culture used as inoculum for experiments was cultivated for approximately 2 months (*Dhc* ∼ 10^6^ cells/mL) from a dormant culture stored at 4 °C for 2 years without feeding, and limited dechlorination activity (i.e., formed VC but not ethene) (Section S2; Figure S6). A total of 12 experimental bottles (and 1 killed control bottle; Figure S7) containing 93 mL sterile RAMM were each inoculated with 7 mL of SDC-9 culture. Perchloric acid (770 μL) was added as the microbial poisoning agent to the killed control bottle prior to inoculation (final pH ∼ 7 after adding acid). Duplicate live control bottles contained no added materials. Another set of duplicate live controls contained 2 g/L sand as a surface control to contrast with biochar. Duplicate treatments contained biochar (2 g/L; 150–250 μm size range; Char350, Char500, Char700, or Char900). The 2 g/L biochar dose was chosen to minimize sorption of PCE, while also providing adequate surface area for microbes to attach and potentially form biofilms. This PCM concentration falls on the low end of the colloidal activated carbon dose range (1.0–36 g/L).^36,37^

Each bottle contained 100 mL of RAMM medium. Cultures were fed lactate (0.15 mL of 60% w/w syrup; 1068 μmol) every 4 days and neat PCE (10–15 μL; 97.6–146.4 μmol per bottle). Initially, 97.6 μmol PCE was added per bottle to controls, Char350, and Char500 treatments, while 146.4 μmol PCE was added per bottle to Char700 and Char900 treatments because of the differential PCE sorption behavior of Chars. After adding sand or biochar and equilibrating for 1 day, the initial PCE mass (aqueous + gas phases) in each bottle was measured before inoculating with SDC-9. An extra 48.8 μmol PCE per bottle was added to Char350 and Char500 treatments on day 46. All bottles were incubated at 23 °C with shaking (100 rpm) in the dark. For comparability, treatments were operated until >80% of the 146.4 μmol PCE added was reduced to ethene. This occurred at different times in different treatments—after 52 days (Char500), 64 days (Char 350), 78 days (Char 700), and 81 days (Char 900). The no-material and sand controls were stopped on day 82.

Experiments were also conducted using highly active dechlorinating cultures (*Dhc* > 1 × 10^11^ cells/mL) with various materials. These included controls without any material, with the four biochars described above (Char350–Char900), with two types of activated carbon (Filtrasorb 200 and Filtrasorb 400, Calgon Carbon), and with sand. In contrast to the dormant culture, the active SDC-9 culture completely dechlorinated PCE to ethene before inoculation and was maintained in RAMM medium supplemented with yeast extract and vitamin B12 in experiments to support high dechlorination activity. More details on these experiments can be found in Section S2, and data are shown in Figures S11 and S12.

### Chlorinated Ethenes, Ethene, and Organic Acid Analyses

Chlorinated ethenes (PCE, TCE, *cis*-DCE, and VC), ethene, and methane were analyzed by injecting headspace samples (100 μL) into an Agilent 6890 GC equipped with a Supelco 1% SP-1000 on a 60/80 Carbopack B column (6 ft × 1/8 in. diameter) and a flame ionization detector (FID) at 250 °C, supplied with hydrogen (40 mL/min) and air (450 mL/min). Nitrogen (30 mL/min) was the carrier gas. Retention times and GC separation conditions are provided in Table S1.

VC, ethene, and methane analytical standards were prepared in serum bottles (30 mL) with known volumes of gas added (Table S2a). PCE, TCE, and *cis*-DCE analytical standards were developed in serum bottles (160 mL) containing 100 mL DI water (Table S2b). Dimensionless Henry’s Law constants (*H*_c_) at 23 °C for PCE (0.64), TCE (0.35), *cis*-DCE (0.14), VC (1.01),^38^ methane (27.03),^39^ and ethene (7.24)^40^ were used to estimate aqueous concentrations and chlorinated ethenes (CEs) + ethene (CE + E) mass balances (aqueous + gas phase) in bottles.

Sorption equilibrium experiments were performed in duplicate 160 mL bottles with 100 mL RAMM, biochar (0.2 g), and known masses of ethene, VC, *cis*-DCE, TCE, and PCE (Table S3). Bottles were incubated at 23 °C with shaking (100 rpm) in the dark. CE concentrations (*C*_e_; μmol/L) were measured by GC-FID after 1 month of equilibration, and the solid phase concentrations (*C*_s_; μmol/g) and partition coefficients (*K*_d_ (L/g)) were estimated using a mass balance approach (Table S3). CE + E mass balances, which include the effect of sorption using estimated *K*_d_ values, are shown in Figure S8. Ethene and methane production rates were estimated from the slope of a linear regression line of measured ethene or methane concentrations over time in bottles (Figures S9 and S10; Table S4).^41,42^

Organic acids (lactate, acetate, propionate, and butyrate) were analyzed by ion chromatography coupled with a conductivity detector in filtered (0.2 μm) liquid samples (1 mL),^41^ as shown in Figure S13. An electron balance of organic acids, CEs, ethene, and methane measured on day 46 and a partial balance for CEs, ethene, and methane measured at the final time points are shown in Table S5.

### Nucleic Acid Extraction, Purification, Quantitation, and Quantitative PCR (qPCR)

Two liquid culture samples (2 mL) were taken from the inoculum for the dormant culture experiment to extract DNA for use as the initial samples (day 0 sample) at the start of the study. Liquid culture samples (2 mL) were taken periodically from experimental bottles during the experiment for DNA extraction. Final liquid (13–14 mL) and solid (biochar or sand; 200 mg dry mass) samples for DNA and RNA extraction were collected on day 52 (Char500), day 64 (Char350), day 78 (Char700), day 81 (Char900), and day 82 (sand; no-material controls (liquid only)). Biochar or sand particles (200 mg dry mass) were separated from the liquid by pouring through a UV-sterilized coffee filter. DNA and RNA extraction and purification procedures for both liquid and solid samples were described previously^43^ and in Section S3. DNA concentrations were measured with the Qubit 4 Fluorometer (Invitrogen by Thermo Fisher Scientific) DNA high-sensitivity assay kit before qPCR.

**Figure 1:**
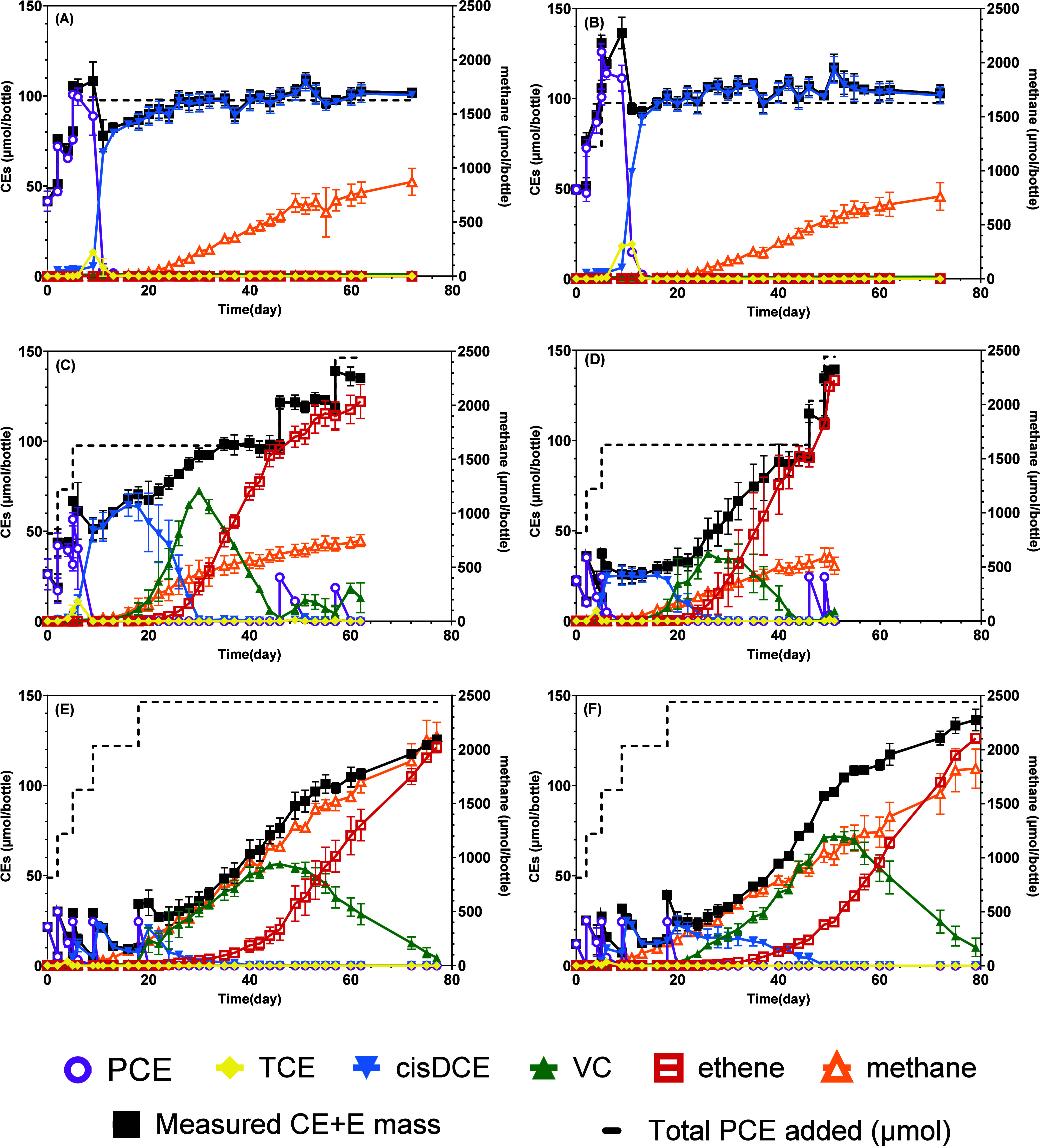
Changes in the mass per bottle (aqueous + gas phase) of methane, chlorinated ethenes (CEs; PCE, TCE, *cis*-DCE, and VC), and ethene (E) with time in PCE-fed SDC-9 cultures. Dotted black lines show the estimated PCE mass spiked into bottles. The measured CE + E mass balance in each bottle is plotted (black squares). (A) SDC-9 without materials; (B) SDC-9 with sand; (C) SDC-9 with Char350; (D) SDC-9 with Char500; (E) SDC-9 with Char700; and (F) SDC-9 with Char900. Data points are the average of duplicate bottles, and the error bars show the complete range of duplicates (maximum and minimum values). Data points that were below the detection of the instrument are shown as 0.

Primers that amplify *Dehalococcoides* (*Dhc*) 16S rRNA genes (DHC-793F/DHC-946R),^44^ *vcrA* (vcr1022F/vcr1093R),^29^ *tceA* (tceA500*F*/795R),^45^ *Desulfitobacterium pceA* (hereafter *Dsf-pceA*) (rdhA29_1488F/rdhA29_1547R),^46^ and *mcrA* (*mcrA*_F3/*mcrA*-rev)^47^ were used for qPCR (Table S6) on DNA extracted from both liquid and solid samples (sand and biochar). Biomarker genes used in this study are further described in Section S3. Synthetic nucleotide “gBlocks”, obtained from Integrated DNA Technologies (IDT; Coralville, IA), containing partial *Dhc* 16S rRNA, *vcrA* (397 bp), *tceA* (318 bp), *Dsf-pceA* (139 bp), and *mcrA* (331 bp) genes (Table S6) were used as qPCR standards. Additional qPCR QA/QC details that follow MIQE guidelines^48^ are provided in Table S7. Gene copies/mL culture and gene copies/g biochar calculations are described in Section S3.

### RNA-Seq and DNA-Seq

Total RNA sequencing (RNA-seq) and whole genome sequencing (DNA-seq) were performed on nucleic acids extracted^43^ on the final sampling days. RNA-seq libraries were rRNA-depleted using probes developed from abundant microbial community members (Section S4; Table S8). RNA-seq and DNA-seq were not performed on sand samples because RNA was not recovered, and DNA recovery was too low for DNA-seq. The number of raw reads from RNA-seq (18 samples sequenced; Table S9) ranged from 39,154,854 to 66,681,063, and from DNA-seq (9 samples sequenced; Table S9) ranged from 42,164,008 to 53,558,356 per sample.

### Recovery of Metagenome-Assembled Genomes (MAGs) Containing Reductive Dehalogenase and Methyl-Coenzyme M Reductase Genes

Quality control of raw reads, contig assembly, and binning methods were adopted from previous work,^43,49^ with details in Section S5. MAGs and unbinned contigs containing reductive dehalogenase (RDase; *rdhA*) and methyl-coenzyme M reductase (*mcrA*) genes are described in Table S9. The *rdhA* and *mcrA* sequences were further verified with an HMMER (version 3.3.2)^50^ hmmsearch against the HMM model for PF13486 (reductive dehalogenase domain), and PF02249 and PF02745 (methyl-coenzyme M reductase alpha subunit domain) downloaded from Pfam.^43,49,51^

### Gene Expression Levels and Differential Gene Expression Analyses

An index of coding DNA sequences (CDS) and Prokka-identified rRNA and tRNA sequences from all MAGs, built with kallisto (version 0.46.1),^52^ was used to map genes in RNA-seq reads, determine effective gene length, and calculate transcripts per million (TPM) values (except rRNA and tRNA) for individual genes and MAGs.^49^ TPM is a commonly used metric in metatranscriptomics to quantify gene expression levels. Details about TPM value calculations are provided in Section S6. Gene counts (except rRNA and tRNA) were used in differential expression (DE) analyses with DeSeq2 (version 3.54.2).^53^

### Correlation of Ethene and Methane Formation Rates with Material and Microbial Properties

Ethene and methane production rates were Spearman’s rank-correlated with material properties and potential growth-indicating genes from *Dehalococcoides* using corrplot (version 0.92)^54^ in R (version 4.3.2).^55^ Variables significantly correlated with ethene production rate were subjected to a principal coordinate analysis (PCA) using R package vegan (version 2.6-10).

## Results and Discussion

### Biochar Properties

The physiochemical properties of biochar pyrolyzed under different temperatures displayed variations in elemental composition, atomic ratio, surface area, pore volume, pore size, conductivity, and electron-donating capacity (Table 1). Consistent with previous studies,^35,56^ we observed increased biochar carbon content (C) as pyrolysis temperature increased. In contrast, the biochar oxygen content (O) and the atomic ratios of H/C and O/C decreased with increasing temperature. Pyrolysis temperature had a prominent effect on biochar surface area, which increased from 16.8 to 261 m^2^/g when pyrolysis temperature increased from 350 to 500 °C. The decrease in biochar surface area when temperature increased from 500 to 700 °C may be explained by the collapse of mesopores.^57,58^ However, further increasing pyrolysis temperature to 900 °C increased surface area to 449 m^2^/g, which might be attributed to the continuous development of micropores.^59^ Surface area measurements using CO_2_ as the adsorbate supported the nitrogen measurements, where the micropore surface area increased from 245 to 543 m^2^/g as pyrolysis temperature increased from 350 to 900 °C. A similar trend was observed for biochar pore volume. Specifically, the total pore volume of biochar increased from Char350 to Char500, which then decreased at Char700 and further increased for Char900. In contrast, both the biochar micropore volume and conductivity increased as temperature increased, while the pore size decreased with increasing pyrolysis temperature (Table 1). The surface area, pore volume, and conductivity of the chars used here and their trends with pyrolysis temperature are consistent with previous work.^59^ The EDC was highest for Char700, which can be explained by the formation of redox-active functional groups.^60–62^

**Table 1:** Elemental Composition, Atomic Ratio, Surface Area, Pore Volume, Pore Size, Conductivity, and Electron-Donating Capacity of Biochar Used in This Study

	elemental composition (wt %)					atomic ratio (mol/mol)									
biochar	C	H	O	N	ash	H/C	O/C	BET SA (m^2^/g)^a^	micro-pore SA (m^2^/g)^b^	total pore volume (cm^3^/g)^c^	micropore volume (cm^3^/g)^b^	pore size (nm)^c^	conductivity (S/m)	EDC (mmol_e–_ /g_char_)^d^	ζ potential (mV)^e^
Char350	72.01	3.43	15.05	1.21	4.75	0.57	0.16	16.8	245	0.01	0.07	5.47	≤0.05	0.26	–35.3 ± 3.4
Char500	77.21	2.59	12.35	1.17	5.80	0.40	0.12	261	331	0.28	0.1	4.34	≤0.04	1.22	–32.7 ± 3.6
Char700	82.99	1.61	7.25	0.87	6.95	0.23	0.07	181	418	0.12	0.13	2.57	32.3	2.03	–14.3 ± 1.1
Char900	80.16	1.09	7.80	1.01	9.50	0.16	0.07	449	543	0.29	0.19	2.58	154.4	1.51	–15.2 ± 5.4

a Surface area (SA) calculated by the Brunauer–Emmett–Teller (BET) theory using N_2_ (77K) from 0.0125 to 0.98 *p*/*p*_0_ using the adsorption branch.

b Calculated by the grand canonical Monte Carlo (GCMC) model, assuming slit pore geometry using CO_2_ (273 K) with up to 0.03 *p*/*p*_0_.

c Calculated by Barret, Joyner, and Halenda (BJH) theory.

d Electron-donating capacity (EDC) calculated with a potentiometric titration method (Section S1; Figure S5).

e Values reported were measured at pH 7 (Figure S4).

#### Biochar Enhances Complete PCE Reductive Dechlorination, Ethene Production, and Methanogenesis by Dormant SDC-9 Cultures Compared to Sand or Absence of a Material

We hypothesized that poplar biochar would accelerate the revival of OHRB in a dormant SDC-9 culture that was stored at 4 °C for more than 2 years without feeding. Live controls (without materials or with sand) reduced 97.6 μmol PCE to TCE and *cis*-DCE within 10 days. The measured chlorinated ethene + ethene (CE + E) (aqueous + gas phase) mass balance in live controls without materials decreased initially during PCE dechlorination and then steadily increased to match the amount of PCE added (Figure 1A). PCE concentration in the killed control continuously decreased (35.6% abiotic PCE loss) with time, but CE dechlorination products were not observed (Figure S7). Apparent PCE losses in both the killed control and the live controls could be explained by PCE sorption onto butyl rubber stoppers and subsequent PCE desorption from the stoppers as dechlorination proceeded in live controls.^63^ The CE + E mass balance in live controls with sand was similar to live controls without materials, indicating that there is comparatively little to no CE sorption on sand surfaces.

Live controls (without materials and with sand) “stalled” at *cis*-DCE for the remainder of the experiment (72 days; Figure 1A,B). In contrast, in the presence of poplar biochar (0.2 g), the dormant cultures reduced *cis*-DCE to VC and ethene (Figure 1C–F). This indicates that poplar biochar accelerated the revival of OHRB and promoted complete PCE dechlorination in a previously dormant SDC-9 culture compared to live controls without biochar or with sand (Figure 1A,B). The dormant seed culture used to inoculate all experimental bottles reduced a portion of *cis*-DCE to VC (Figure S6), indicating that *Dhc* was active at the beginning of the experiment. However, because the PCE mass added per 100 mL bottle was normalized across all treatments, the diluted inoculum in live controls experienced higher initial aqueous PCE concentrations (∼162 mg/L) than the seed culture (∼40 mg/L). These PCE concentrations approach the aqueous solubility limit of PCE and exceed reported thresholds known to inhibit *Dehalococcoides* growth (e.g., >70 mg/L PCE).^22,64^ PCE toxicity likely contributed to the lack of further *cis*-DCE reduction in the live controls (no materials and sand). The absence of complete CE dechlorination to ethene under the relatively high PCE loadings in live controls with sand suggests that surfaces capable of sufficiently sorbing CEs are required to lower aqueous levels and alleviate toxicity to OHRB such as *Dhc*.

Indeed, all four biochars sorbed CEs and ethene (CE + E) to varying extents, as shown by estimates of CE and ethene partitioning coefficients (*K*_d_) (Table S3). In general, Char700 and Char900 sorbed more CE + E than Char350 and Char500. *K*_d_ was highest for PCE (36 ± 5.9 L/g) and TCE (31 ± 3.7 L/g) in Char900, and lowest for PCE (1.4 ± 0.5 L/g) and TCE (0.8 ± 0.1 L/g) in Char350. *K*_d_ values for *cis*-DCE, VC, and ethene were also lower in Char350 and Char500 (ranging from 1.2 ± 0.1 to 0.1 ± 0.0 L/g) than in Char700 and Char900 (ranging from 5.2 ± 0.0 to 0.2 ± 0.3 L/g; Table S3). CEs will adsorb more strongly to Char700 and Char900 than to Char350 and Char500 (Table S3); thus, equilibrium aqueous-phase CE concentrations will decrease in the presence of chars with increasing pyrolysis temperature.

The impact of CE + E sorption in the biochar treatments can been seen by discrepancies between the measured CE + E (aqueous + gas phase) mass balances and the estimated total amount of PCE added (Figure 1C–F). These discrepancies, which increased with pyrolysis temperature, are attributed to sorption of PCE onto biochar. Apparent increases in measured CE + E mass during the experiment could reflect gradual desorption of sorbed PCE (and other CEs produced during dechlorination) from biochar and subsequent dehalogenation to ethene. Calculated CE + E mass balances (aqueous + gas + solid phases), performed by estimating sorbed CE mass using *K*_d_ values measured in separate abiotic equilibrium sorption tests for each char (Table S3), provided an explanation for the observed missing CE + E mass, particularly as PCE desorbed from chars and was dechlorinated to less sorptive VC and ethene (Figure S8). This *K*_d_-based approach should improve mass balance closure, but discrepancies remain because sorption equilibrium is assumed. Live cultures with active OHRB that also contain sorbents are dynamic, with CEs being produced and consumed continuously, and sorption/desorption processes that can exhibit hysteresis and nonequilibrium. As a result, true sorption equilibrium may not be reached at every sampling time point. Larger discrepancies in calculated CE + E mass balances in the Char700 and Char900 treatments indicated that a fraction of PCE may remain associated with the biochar without a measurable aqueous-phase concentration, increasing uncertainty in *K*_d_-based sorbed-mass estimates when aqueous concentrations approach detection limits. Therefore, Figure S8 is intended to show sorption trends (e.g., greater retention of sorbed PCE on Char700/Char900), rather than a mass balance closure at every sampling point. Overall, agreement between the PCE mass added and the calculated CE + E mass tends to improve as CE dechlorination proceeds to less sorptive products (i.e., VC and ethene).

Ethene production rates between days 28 and 44 averaged 5.1 μmol/bottle/day (Char350 treatment) and 5.6 μmol/bottle/day (Char500 treatment) and decreased to 1.7 μmol/bottle/day (Char350 treatment) or increased to 7 μmol/bottle/day (Char500 treatment) after adding PCE on day 46 (Figure S9; Table S4). Ethene production rates averaged 3.2 μmol/bottle/day (Char700 treatment) and 3.7 μmol/bottle/day (Char900 treatment) after 46 days (Table S4). The higher initial ethene production rates observed for Char350 and Char500 are consistent with their lower adsorption affinity for PCE compared to Char700 and Char900 (*p* = 0.0012), which would allow for higher aqueous PCE and daughter-product availability. In contrast, PCE additions after day 46 decreased the ethene production rate in the Char350 treatment but increased the ethene production rate for the Char500 treatment. The higher surface area of Char500 compared to Char350 (i.e., 261 vs 16.8 m^2^/g) may provide additional PCE adsorption sites. As a result, the Char350 treatment experienced higher aqueous CE concentrations that might have exerted toxicity to dechlorinating microbes,^65^ resulting in the ethene production rate decreasing from 5.1 to 1.7 μmol/bottle/day.

These observations indicate a nonmonotonic relationship between biochar CE sorption affinity and revival of complete CE dechlorination in stressed SDC-9 cultures. Under the relatively high PCE loadings used in this study, some sorption is required to lower aqueous PCE concentrations to noninhibitory levels, as evidenced by the stalled *cis*-DCE reduction in the no-material and sand controls compared to the biochar treatments. However, the higher-temperature chars (Char700 and Char900), which exhibited greater sorption capacity, likely sequestered PCE and its daughter products more strongly, thereby reducing their aqueous bioavailability and contributing to the lower ethene production rates observed relative to Char350 and Char500. Thus, Char350 and Char500 appear to strike a balance between mitigating PCE toxicity and maintaining sufficient aqueous CE bioavailability, suggesting that an intermediate sorption regime is most favorable for sustaining complete dechlorination under our experimental conditions. Future experiments with and without biochar across a range of initial PCE concentrations would help more clearly distinguish CE sorption effects from toxicity. Nevertheless, our results support the idea that sorption to biochar reduces aqueous PCE to less inhibitory levels, enabling revival of complete CE dechlorination in dormant SDC-9 cultures.

However, the different reactivity of chars toward CE dechlorination cannot be attributed solely to adsorption. Although the *K*_d_ values of PCE increased from Char350 to Char900—most notably with a nearly 10-fold rise from Char700 to Char900 (*K*_d_ of 3.9 vs 36 L/g)—the corresponding changes in ethene production rates were minimal (3.2 vs 3.7 μmol/bottle/day). These findings suggest that the influence of char physicochemical properties on microbial dechlorination is more intricate than adsorption affinity alone, as indicated by *K*_d_ values. Additional effects such as enhanced cell attachment or microscale redox gradients might also contribute. A deeper investigation of the effects of other biochar characteristics on CE dechlorination by SDC-9 is essential to uncover the underlying mechanisms.

Methanogenesis was observed in all controls and treatments (Figure 1). Methane production rates in live controls and in the Char350 and Char500 treatments were between 16 and 26 μmol/bottle/day, while methane production rates in the Char700 and Char900 treatments were higher (34 and 26.5 μmol/bottle/day) (Figure S10; Table S4). Because methanogens use the same electron donor as many OHRB (hydrogen), increased methanogenesis rates could lower the electron donor levels available for CE dechlorination^66,67^ and partially explain the slower ethene production rates in Char700 and Char900 treatments compared to the Char350 and Char500 treatments (Table S4).

We investigated the impact of sand and biochar on ethene and methane production rates in highly active SDC-9 cultures to provide a high-activity comparison to revival experiments with dormant SDC-9 cultures and evaluate whether biochar influences CE dechlorination under growth conditions where OHRB and methanogens are not stressed (e.g., by starvation). We hypothesized that poplar biochar would enhance PCE dechlorination and ethene formation rates in these cultures, compared to ethene formation rates in no-material and sand controls. All biochar treatments completely dechlorinated 97.6 μmol of PCE to ethene within 17 days (Figure S11C–F), whereas the no-material and sand controls dechlorinated 97.6 μmol of PCE in 32 days (Figure S11A,B). Ethene production rates were highest in the Char500 treatment (6.8 μmol/bottle/day), compared to Char350 (5.2 μmol/bottle/day; *p* = 0.01), Char700 (4.6 μmol/bottle/day; *p* = 0.007), and Char900 (5.3 μmol/bottle/day; *p* = 0.087) (Table S4). Ethene production rates in biochar treatments were higher than those in the sand (2.0 μmol/bottle/day; *p* = 0.0008) and no-material (2.6 μmol/bottle/day; *p* = 0.0027) controls. These findings indicate that poplar biochar promotes complete PCE dechlorination in highly active cultures compared to controls with no materials or sand.

We also evaluated the impact of granular activated carbon (specifically Filtrasorb200 (AC200) and Filtrasorb400 (AC400)) on PCE dechlorination and ethene formation rates in highly active SDC-9 cultures. PCE (97.6 μmol) was completely dechlorinated to ethene within 12 days in all activated carbon treatments (Figure S12). Ethene production rates in the AC200 (4.6 μmol/bottle/day) and AC400 (4.1 μmol/bottle/day) treatments were higher than those in the sand (2.0 μmol/bottle/day; *p* < 0.013) and no-material (2.6 μmol/bottle/day; *p* < 0.034) controls, but not significantly different from the biochar treatments, except for Char500 (*p* < 0.013). CE sorption behavior in the activated carbon treatments was similar to Char 900 treatments, as evidenced by discrepancies between the mass of PCE added and the CE + E mass balance near the end of the experiment (Figure S12), suggesting that CEs (i.e., PCE, TCE, and/or *cis*-DCE) remain sorbed to activated carbon.

#### Electron Balance of Ethene, Methane, and Fermentation Products and Gene Expression Associated with Fermentation Reactions

Lactate spikes (1068 μmol; 0.013 electron equivalents (eeq)) were consumed rapidly and fermented to acetate, propionate, and butyrate in all bottles (Figure S13). An electron balance at day 46 (Table S5; Figure S14) shows that 2.7–10.9% (acetate), 1.3–6.2% (propionate), and 46–55% (butyrate) of the lactate eeq were in volatile fatty acids (VFAs). Acetate serves as a carbon source for obligate OHRB such as *Dhc*.^68,69^ The remaining electrons were distributed among measured products (ethene and methane) and other unmeasured products (e.g., biomass, hydrogen, and other microbial processes). Although H_2_ was not measured in this study, the standing H_2_ pool was likely small because of rapid consumption by OHRB and methanogens, and electrons that transiently passed through H_2_ are reflected in the methane and ethene eeq.

Expression of genes encoding enzymes involved in key fermentation reactions was observed in biochar-attached and suspended cells in all treatments (Char350, Char500, Char700, and Char900) and in the no-material control (Figure S16). These reactions include lactate degradation; acetate, propionate, and butyrate formation; hydrogen consumption (indicated by hydrogenase gene expression from *Dhc*, *Desulfitobacterium* and methanogens); and hydrogen production (hydrogenase gene expression by other microbes in SDC-9 that are not OHRB or methanogens). Both OHRB (e.g., *Dhc* and *Desulfitobacterium*) and hydrogenotrophic methanogens present in SDC-9 use and compete for hydrogen as an electron donor.^66^ Although hydrogen generated during lactate fermentation was not measured directly in this experiment, indirect evidence of its consumption can be inferred from hydrogenase gene expression in known hydrogen consumers (i.e., *Dehalococcoides*, methanogens, and *Desulfitobacterium*) (Figure S16). The specific flow of electrons from hydrogen and the competition for hydrogen between OHRB and methanogens cannot be quantified from our data. Future work that includes direct hydrogen measurements and isotope-based tracing approaches would shed light on these microbial interactions.

Since lactate was provided in quantities far exceeding the electron demand for CE dechlorination, only a small fraction of the total lactate eeq was utilized for CE dechlorination and ethene formation. More electrons from lactate were used for ethene production (0.5–0.6% eeq) in Char350 and Char500 treatments than in Char700 and Char900 treatments (0.1–0.2% eeq). There were no eeq in ethene in controls at day 46 or by the end of the experiment. However, the % of electrons from lactate used to form ethene increased in Char700 and Char900 treatments by the end of the experiment (0.4% eeq on days 77–79). Conversely, more electrons from lactate were used for methanogenesis in Char700 and Char900 treatments (5.1–6.2% eeq) than in Char350 and Char500 treatments (3.0–3.5% eeq) on day 46 and at the end of the experiment (6–7% eeq for Char700/900 vs 2.7–2.9% eeq for Char350/500) (Table S5; Figure S14).

#### Growth of OHRB and Methanogens during PCE Dechlorination in the Presence and Absence of Biochar

SDC-9 contains different OHRB that reduce PCE to *cis*-DCE (*Desulfitobacterium* sp.; *Dsf*), and *cis*-DCE to VC and ethene (*D. mccartyi*; *Dhc*) using reductive dehalogenases encoded by the genes *Dsf-pceA*, *tceA*, and *vcrA*.^9,43,70,71^ We monitored *Dhc* biomarkers (*Dhc* 16S, *tceA,* and *vcrA)* that indicate *cis*-DCE transformation to ethene in SDC-9, as well as *Dsf* biomarkers (*Dsf-pceA*) that indicate reduction of PCE to *cis*-DCE in SDC-9. The initial *Dhc* 16S, *tceA*, and *vcrA* abundance in each bottle (∼10^5^ copies/mL) did not change in live controls (i.e., no materials and sand), indicating that *Dehalococcoides* did not grow when *cis*-DCE was not reduced to VC or ethene, consistent with the observed stall at *cis*-DCE (Figure S15A,B). The *Dsf-pceA* abundance in no-material and sand controls increased from 10^5^ to 10^6^ copies/mL, consistent with growth-coupled PCE reduction to *cis*-DCE by *Dsf* (Figure S15A,B).

In biochar treatments, *Dsf*-*pceA* abundances rose by about 1 order of magnitude over the first 17 days, and *Dhc* 16S, *vcrA*, and *tceA* abundances rose by about 2 orders of magnitude over 64 days, consistent with initial growth of *Desulfitobacterium* with PCE as the electron acceptor, followed by growth of *Dehalococcoides* with increased abundance of *tceA* and/or *vcrA* with *cis*-DCE and VC as the electron acceptor in the presence of poplar biochar (Figure S15). In the Char350, Char700, and Char900 treatments, *Dsf-pceA* abundance declined after day 17 (Figure S15), which is consistent with declining availability of PCE in the aqueous phase due to consumption and sorption (Figure 1). In contrast, this *Dsf-pceA* abundance decline was not observed in the Char500 treatment (Figure S15D). The sharper decline in *Dsf-pceA* abundance in the Char350 treatment than in the Char500 treatment indicates that fewer *Dsf* were present to dechlorinate PCE when more PCE was added on day 46 (Figure 1). This pattern is consistent with the decrease in ethene production rates observed in Char350 treatment after day 46 (Figure S9; Table S4), while the rates were less affected in the Char500 treatment.

Attachment of *Dsf* to biochar is indicated by *Dsf-pceA* abundances on biochar solids at about 10^7^ copies per bottle across all biochar treatments between days 52–81 (Figure 2). In the Char500 treatment, *Dsf-pceA* levels were 2.5 times higher than in Char350, 5 times higher than in Char700, and 3 times higher than in Char900, but there was no clear monotonic trend with pyrolysis temperature. In the sand treatment, *Dsf-pceA* copies attached to sand were about 10^3^ copies per bottle, indicating much less attachment to sand than to any of the biochars.

**Figure 2:**
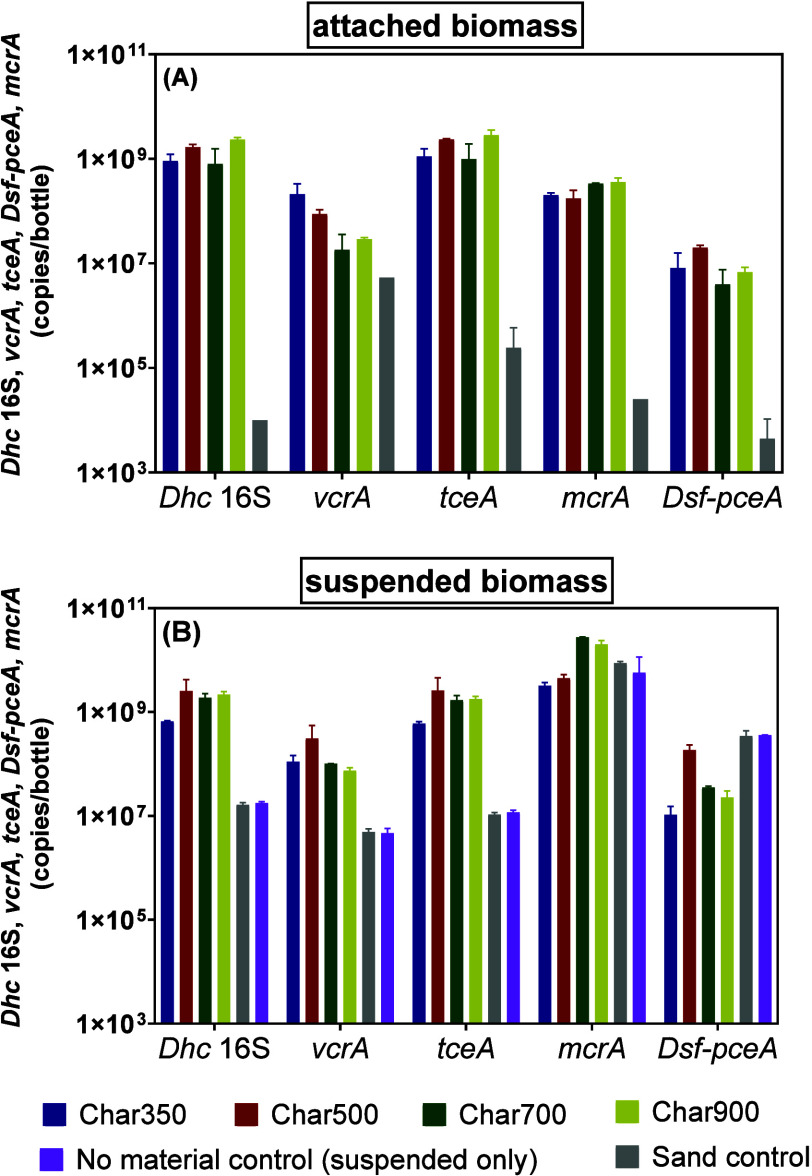
Abundance of CE dehalogenation biomarkers (*Dhc* 16S, *vcrA*, *tceA*, and *Dsf-pceA*) and methanogenesis (*mcrA*) biomarkers in (A) biochar samples (0.2 g) and (B) liquid samples after dechlorinating 146.4 μmol PCE. Data points represent the average of two replicates bottles, and the error bars are the complete range of duplicates (maximum and minimum values). Gene copies/bottle calculations for both attached and suspended biomass are described in Section S3. The gene quantification limit for suspended biomass is 1.5 × 10^5^ copies/bottle and for attached biomass is 3.0 × 10^3^ copies/bottle.

*Dhc* also attached to biochar, as indicated by *Dhc* 16S and *tceA* abundances on biochars between days 52 and 81 ∼10^9^ copies/bottle in all biochar treatments (Figure 2). The *vcrA* abundance of attached biomass was 0.7–0.8 orders of magnitude lower than *Dhc* 16S and *tceA* abundances in Char350 and Char500 treatments and 1.4–1.6 orders of magnitude lower in Char700 and Char900 treatments (Figure 2). Only the abundance of *vcrA* on biochar showed a general decrease with increasing pyrolysis temperature, with a small increase at Char900, whereas attached *Dhc* 16S and *tceA* copy numbers varied among chars without a consistent trend. Only one replicate showed *Dhc* attached to sand (10^4^ *Dhc* 16S gene copies/bottle), which was 4.4–5.4 orders of magnitude lower than in biochar treatments (Figure 2), indicating that *Dhc* attached less to sand compared to biochar. The 0.7–0.9 order of magnitude lower *vcrA* abundances in Char700 and Char900 treatments compared to the Char350 and Char500 treatments suggests that *Dhc* harboring *vcrA* attached more on the Char350 and Char500 surfaces.

Methanogens grew in all live controls and biochar treatments, as evidenced by increasing suspended culture *mcrA* abundance in all bottles from an initial abundance of ∼10^6^ gene copies/mL between days 0 and 40 until reaching the stationary phase at 10^7^–10^8^ gene copies/mL after 40–60 days (Figure S15). The *mcrA* abundance on biochars between days 52 and 81 was about 10^8^ copies/bottle in all treatments, which indicated that methanogens attached to biochar (Figure 2), as seen previously.^72^ In the Char700 and Char900 treatments, *mcrA* abundance was 0.3 orders of magnitude higher in attached cells and 0.8 orders of magnitude higher in suspended cells compared to the Char350 and Char500 treatments. This suggests that methanogens were more enriched on the surfaces of Char700 and Char900, which is consistent with the higher methane production observed in these treatments.

Microscopic imaging was beyond the scope of this study, so the detailed biofilm structure of *Dhc*, *Dsf*, and methanogens on biochar surfaces remains unresolved. Future experiments using techniques such as fluorescence in situ hybridization (FISH) and geneFISH could be used to directly visualize attached *Dhc*, *Dsf*, and methanogens on biochar surfaces.

#### Recovery of MAGs and Overall Gene Expression Relevant to Dehalogenation and Methanogenesis

Four MAGs and two unbinned contigs containing reductive dehalogenase genes and four MAGs classified as methanogens were obtained from SDC-9 cultures (Table S10). Only the unbinned vcrA_contig contains *vcrA* found in *Dhc*, which participates in ethene production.^43^ However, the vcrA_contig was not binned with the *Dhc* MAG, although >70% of its nucleic acid sequence was more than 98% identical with several other *Dhc* strains. This implied that the *Dhc* strain harboring the vcrA_contig might be different than the *Dhc* MAG. This was supported by qPCR, where *vcrA* abundance was less than *Dhc* 16S rRNA gene abundance in both liquid and attached phases (Figure S15). Similarly, another unbinned contig contained *Dsf-pceA*, which participates in PCE to *cis*-DCE transformation, with an inferred product sharing 92% amino acid identity with the inferred product from *Desulfitobacterium hafniense*.^73^ Because the unbinned contigs are relevant to chlorinated ethene dehalogenation in SDC-9, they were analyzed along with the MAGs.

From the MAGs and contigs (Table S10), we identified 12 different RDase and 4 different *mcrA* genes and examined their expression levels across all samples (Figure S17). In live controls, there was evidence of low-level (basal) expression of *tceA* and *vcrA*, but *Dsf-pceA* (WRX71687.1) was the most expressed RDase gene, with a TPM 1 order of magnitude greater than *tceA* and *vcrA* (Figure S17). A wider range of RDase genes were expressed in the presence of biochar, particularly *tceA* and *vcrA* (with TPM values 2–5 orders of magnitude greater than the no-material control), irrespective of pyrolysis temperature. In contrast, *mcrA* expression was similar among all treatments and controls, apart from *Methanobrevibacter*.

The disparity between *vcrA* and *Dsf-pceA* expression in the no-material control is consistent with its *cis*-DCE accumulation behavior. The *cis*-DCE and VC accumulation behavior of SDC-9 cultures during the early phases of growth in batch cultures has been attributed to more rapid growth of OHRB that reduce PCE to *cis*-DCE (i.e., *Dsf*) than OHRB that reduce *cis*-DCE to VC and ethene (i.e., *Dhc*),^9^ possibly because of delayed induction of *cis*-DCE reduction genes (e.g., *vcrA*) relative to PCE reduction genes (e.g., *Dsf-pceA*). It is plausible that the stress of long-term cell storage without feeding endured by dormant SDC-9 cultures cause *Dhc* cells to enter a “viable but not culturable” (VBNC) state that was not overcome under our experimental design (except with biochar present).

#### Gene Expression Related to *Dhc* Growth in the Presence of Biochar

Studies with pure *Dehalococcoides* cultures indicate that they lack the ability to de novo synthesize several cofactors required for growth, including vitamin B12, biotin, and thiamine.^74,75^ Because exogenous vitamins were not provided to dormant cultures in our experiments, OHRB like *Dhc* must rely on exogenous sources (e.g., other microbial community members) to obtain these required cofactors. Vitamin B12 is not synthesized by plants;^76,77^ thus, biochar derived from plant biomass, such as poplar tree biomass, is an extremely unlikely source of vitamin B12. Although vitamin B12 is considered heat-stable, its half-life is very short at high temperatures, approximately 80 s at 141.6 °C in aqueous solutions,^78^ our study involved producing biochar through pyrolysis at temperatures ranging from 350 to 900 °C for 2 h, conditions that would likely degrade or destroy any vitamins that may have existed in the plant biomass feedstock. However, expression of genes involved in vitamin B12 biosynthesis and its precursors (i.e., the corrin ring: cobyrinate *a*,*c*-diamide, and the lower ligand: 5,6-dimethylbenzimidazole), biotin, and thiamine, was observed in controls and treatments (Figure S18), suggesting that microorganisms in SDC-9 do indeed synthesize vitamins important for *Dhc* growth, particularly vitamin B12. In the control, expression of genes required for vitamin B12 lower ligand synthesis was less than observed in the treatments, which suggests a possible limiting step for vitamin B12 production that may have delayed *Dehalococcoides* revival in no-material and sand controls during the experiment.

Carbon-based material properties affect quorum sensing molecule production and influence microbial attachment on biochar surfaces (i.e., biofilm formation).^79,80^ Previous studies have postulated that biochar promotes OHRB growth by providing suitable surface conditions for attachment.^23,24^ Our data confirm that *Dhc* attached to biochar surfaces and expressed quorum sensing and biofilm formation genes but were also active in the suspended fraction. Expression of several *Dhc* MAG quorum sensing genes significantly increased (Figure 3), but complete autoinducer production, release, and accepting pathways were not found. In contrast, several *Dhc* biofilm formation genes (*bapA*, *wza*, and *crp*)^81–83^ were expressed in the treatments (Figure 3). *Dhc* cells sometimes exhibit filamentous appendages (i.e., pili), which supposedly facilitate attachment to other cells or surfaces.^74^ Correspondingly, type IV pilus assembly genes (i.e., *pilABCMOTX*) in the *Dhc* MAG were expressed in the treatments (Figure 3). Taken together, these observations suggest that biochar promotes *Dhc* biofilm formation. It is plausible that *Dhc* is initially attracted to and forms biofilms on biochar surfaces, which helps revive ethene production in our experiments, with active cells later releasing into the liquid after biofilm establishment.^81–84^

**Figure 3:**
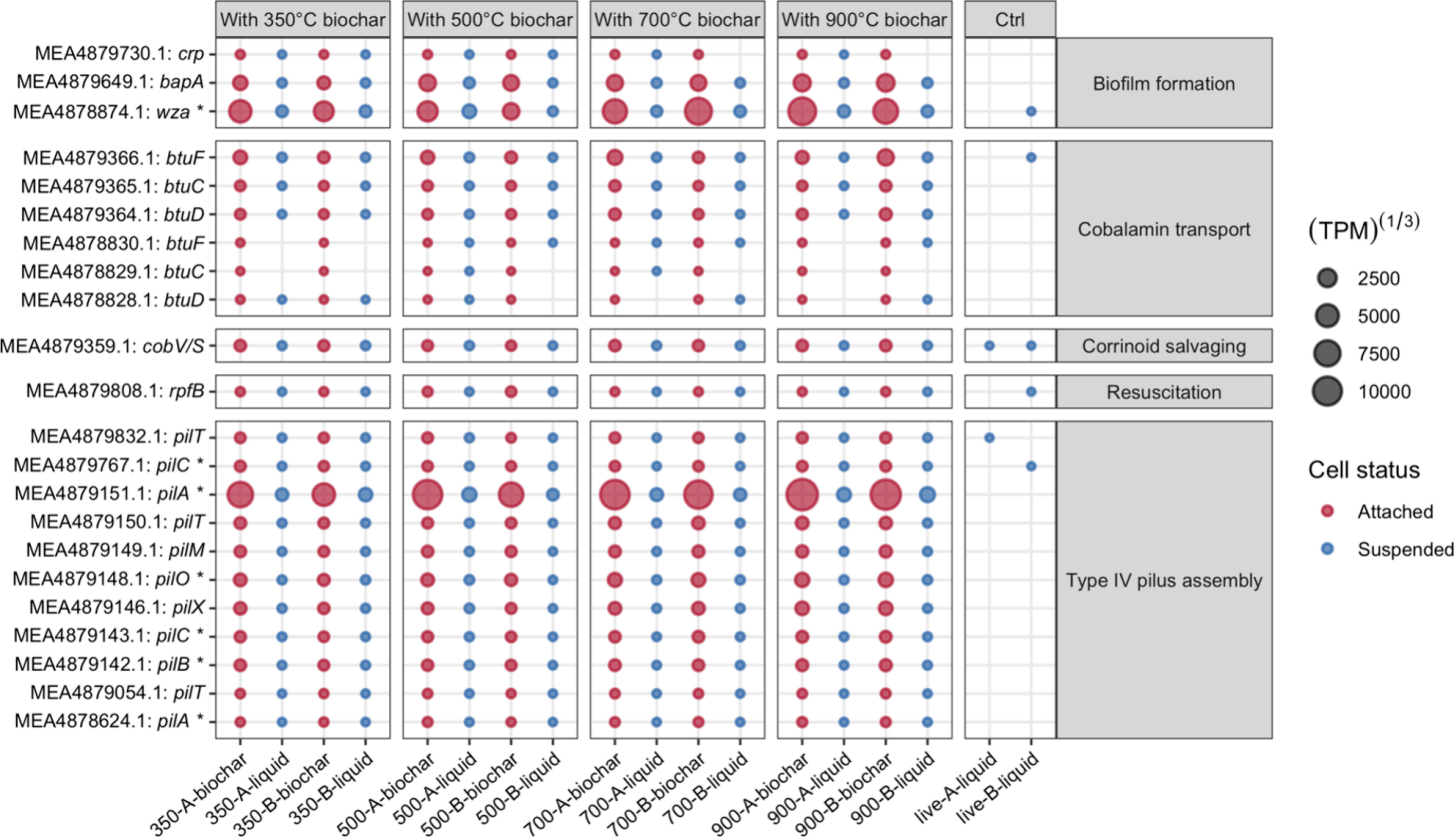
Expression of all *Dehalococcoides* genes related to biofilm formation, cobalamin transport across the membrane, corrinoid salvaging, resuscitation-promoting factor RpfB, and type IV pilus assembly in biochar treatments (both suspended (blue bubbles) and attached (red bubbles) fractions) and in the no-material control (ctrl). The size of the bubble is proportional to the gene expression level (shown in transcripts per million to the 1/3 power (TPM)^1/3^). A “*” after the gene name indicates that gene was only annotated with the KEGG Orthology families database (KOfam). The remaining genes were annotated using both the NCBI Prokaryotic Genome Annotation Pipeline (NCBI-PGAP) and KOfam.

The resuscitation-promoting factor *B* gene (*rpfB*) was significantly expressed in all biochar treatments, but not in the live controls without materials or with sand (Figure 3). RpfB has been implicated in the resuscitation of dormant bacteria and in promoting biofilm formation.^85,86^ Rpf amendment has been shown to improve the growth of *Dhc* in a culture that dechlorinates both PCE and PCBs.^87^ We speculate that RpfB produced by *Dhc* in the presence of biochar plays a role in resuscitating dormant *Dehalococcoides* cells under the conditions of our experiment.

Expression of *btuCDF* from *Dehalococcoides*, the product of which transports cobalamin across cell membranes,^88^ increased with biochar addition (Figure 3), suggesting that *Dhc* in SDC-9 transported cobalamin produced by other microbes.^89,90^ In addition, *cobV/S* from *Dehalococcoides* were also expressed, suggesting that *Dhc* salvaged cobyrinate *a*,*c*-diamide produced by other microbes. However, compared with *cobV/S* expression, the higher expression of *btuCDF* and its absence in the live control suggests that *Dehalococcoides* could obtain vitamin B12 produced by other microorganisms in SDC-9, as previously reported for *D. mccartyi*.^91^ Direct HPLC-MS quantification of cobalamin in future work could be useful to confirm salvage and transport gene expression and to test whether vitamin B12 availability limits *Dehalococcoides* revival in the absence of biochar.

#### Correlations between Ethene/Methane Production Rates and Biological and Physiochemical Properties

A Spearman’s rank correlation analysis was used as an exploratory tool to evaluate potential relationships between ethene and methane production rates, MAG and functional gene abundance and expression data for *Dhc* and methanogens, and physicochemical biochar properties in biochar treatments (Figure S19). Variables significantly correlated with ethene production are summarized in Figure 4. Ethene production rates were positively correlated (*p* < 0.05) with pore size, nitrogen content (i.e., N at % and N wt %), oxygen content (O wt %), O/C atomic ratio, *Dsf-pceA* and *vcrA* abundance in attached cells, and *rpfB* TPM in suspended and attached cells. Conversely, ethene production rates were negatively correlated (*p* < 0.05) with methane production rate, ζ potential, conductivity, EDC, *K*_d_ value for VC and *cis*-DCE, at % C=O, wt % C, total methanogen TPM in suspended cells, *mcrA* abundance in suspended and attached cells, and *wza* and *pilTC* TPM in attached cells.

**Figure 4:**
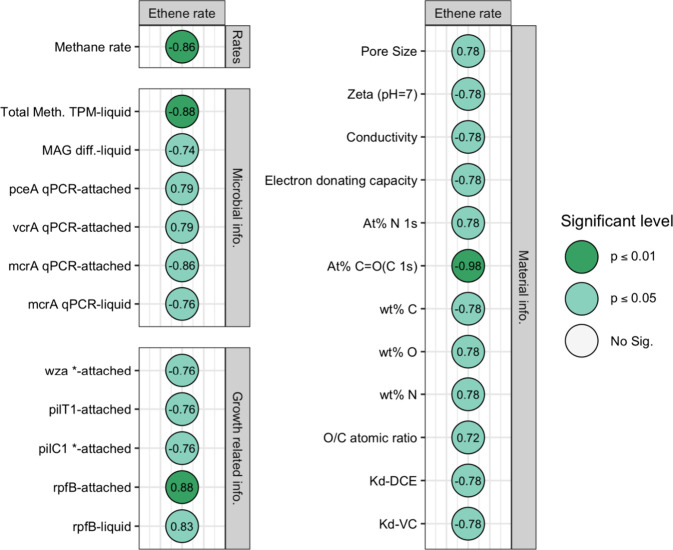
Spearman’s ranking correlation matrix between ethene production rates and microbial and material properties in biochar treatments. Correlation values colored with dark green represent *p* value <0.01, with light green represent *p* value <0.05, with no color represent *p* value >0.05. The suffix-liquid indicates the value from suspended cells, and the suffix-attached indicates the value in biochar-attached cells. MAG diff.: the difference of the TPM of vcrA_contig over the TPM of total methanogens; Meth. TPM: the TPM of methanogens. *wza*: polysaccharide biosynthesis/export protein, *pil* (*pilT*/*pilC*): pilus formation; *rpfB*: resuscitation-promoting factor B; At % represents atom content quantified by X-ray photoelectron spectroscopy. wt % represents the elemental composition quantified by energy dispersive spectroscopy. *K*_d_: partitioning coefficient. A “*” after the gene name indicates that the gene was only annotated with KOfam. The remaining genes were annotated using both the NCBI-PGAP and KOfam.

The Spearman’s correlation matrix also indicates significant cocorrelations (both positive and negative) exist between many of the biochar material properties (Figure S18). A principal component analysis (PCA) was conducted with parameters significantly correlated with ethene formation rate. A PCA biplot further visualizes cocorrelations among parameters (Figure S19). Overall, the variables formed two groups driven by Char350 and Char500 treatments distributed on the left and Char700 and Char900 on the right (Figure S20). For instance, pore size was cocorrelated with variables including wt % N, wt % O, and O/C atomic ratio (correlation coefficient ≥0.7). Since many biochar physicochemical properties varied together with changes in pyrolysis temperature, the correlation analysis cannot identify single controlling biochar properties related to ethene formation rates. This analysis is intended to generate hypotheses for future research rather than provide definitive mechanistic evidence that links biochar properties with impacts on ethene formation rates in SDC-9 cultures.

The increases in surface area and pore volume associated with increases in pyrolysis temperature were not correlated with ethene formation rate (Figure S19). For instance, despite the more than 15-fold increase in surface area and 28-fold increase in pore volume of Char500 compared to Char350, the ethene formation rate in the presence of Char500 increased by 9.8%. In addition, Char900 has the highest surface area and pore volume (Table 1); however, it was less effective in promoting ethene production than Char350 and Char500. This suggests that biochar properties such as surface area and pore volume have minimal influence on SDC-9 ethene formation rates among the treatments.

Conversely, the average biochar pore size was significantly positively correlated with ethene production rate. The effect of pore size on ethene formation rate could be explained by pore-filling and desorption hysteresis mechanisms. Pore filling involves the diffusion of PCE molecules into the narrow micropores (<2 nm) of biochar. Once inside, these molecules are physically constrained by the pore walls, making it difficult for them to diffuse back out. This is especially true for PCE, which has a relatively large molecular size (∼6.6 Å), making it more susceptible to entrapment in tight pore spaces. Wang et al.^92^ observed increasing desorption hysteresis with increasing pyrolysis temperature. The hysteresis index (HI) increased from 0.380 (BCC300) to 0.661 (BCC700), indicating that PCE was more strongly retained in biochars with greater pore-filling capacity.^92^ In addition to average pore size, micropore surface area and micropore volume also increased with pyrolysis temperature (Table 1), indicating a greater abundance of accessible microporous domains for pore filling. We hypothesize that the micropores in biochar play a critical role in trapping PCE, primarily through a pore-filling mechanism that becomes particularly significant when pore sizes approach 2 nm. Collectively, average pore size, micropore surface area, and micropore volume govern PCE confinement and desorption hysteresis, rather than pore size alone. This mechanism, along with desorption hysteresis, may contribute to the delayed release of PCE, especially in high-temperature biochars such as Char700 and Char900. Delayed desorption of PCE from chars would lead to decrease reductive dechlorination rates and hence decreased ethene formation rates. However, these variables are interrelated and mainly follow the same pyrolysis-temperature gradient. Therefore, although pore-filling and desorption hysteresis could explain stronger retention and slower dechlorination in higher-temperature chars, our correlation analysis cannot differentiate pore-size effects from related biochar material properties. Future studies integrating time-resolved kinetic measurements and pore-scale or reactive transport modeling could further elucidate the mechanistic role of pore structure in controlling sorption and biodegradation.

Increasing pyrolysis temperature increased biochar conductivity and EDC and facilitated the dynamic evolution of functional groups on Chars350–900. However, ethene production rates were higher in Char350/Char500 treatments despite lower EDCs and 3–4 orders of magnitude smaller conductivity than Char700 and Char900. This indicates that biochar conductivity and EDC were not the main factors controlling PCE-to-ethene dechlorination by OHRB (e.g., *Dhc*) in our experiments. This result is expected because *Dhc* utilizes hydrogen, generated by fermentation processes, as the physiological electron donor for organohalide respiration.^91,93^ Growth of *Dhc* has not been shown to rely on directly accepting electrons from conductive surfaces such as biochar; instead, it relies on indirect pathways involving other community members.^94,95^ Conversely, methane production rates were higher in the presence of the more conductive and redox-active Chars (Char700 and Char900). The conductivity of biochars is thought to enhance methane production in anaerobic digesters by stimulating direct interspecies electron transfer (DIET) involving methanogens.^96,97^ However, DIET was not directly tested here. Because DIET is difficult to confirm without targeted validation, future work could combine inhibitor tests and direct hydrogen measurements to quantify the contribution of DIET in methanogenic and other electron transfer processes in these mixed cultures.

ζ potential values of all four biochar types were negative at pH 7 (Table 1). For instance, Char350/500 had a less negatively charged surface than Char700/900, which is less repulsive toward negatively charged bacteria or substrates (e.g., lactate) than Char700/900, which could influence the fermenting population in SDC-9.^98^ More studies are needed to elucidate how ζ potential in biochar influences ethene production, methanogenesis, and supporting fermenting populations in anaerobic CE-dechlorinating cultures like SDC-9.

#### Environmental Implications

Declining abundance and activity of OHRB (e.g., *Dhc*) following bioaugmentation or biostimulation during anaerobic CE bioremediation could occur because of substrate scarcity and competition with other microbes (e.g., methanogens) for electron donors and growth factors (e.g., H_2_, cobalamin), leading to decreased dehalogenation and ethene formation rates that could hamper the efficiency of anaerobic bioremediation processes. In this study, we specifically addressed this challenge by showing that a long-starved, low-activity, commercially available PCE-dechlorinating consortium (SDC-9) stalled at *cis*-DCE in no-material and sand controls, while *Dhc* growth was revived and complete PCE dechlorination to ethene was restored in the presence of poplar biochar under these stressed conditions. In highly active SDC-9 cultures, both biochar and activated carbon further enhanced PCE dechlorination, as evidenced by increased ethene formation rates compared to controls. Collectively, these experiments suggest that poplar biochar improves CE dechlorination performance by promoting *Dehalococcoides* growth and biofilm formation.

By producing poplar biochar using a controlled pyrolysis-temperature gradient (350–900 °C) from the same feedstock and batch, we created a set of materials with a gradient of sorption behaviors and physicochemical properties that changed predictably based on pyrolysis conditions. Ethene production rates by SDC-9 cultures differed markedly among the chars in response to this gradient. Char350 and Char500 provided an effective balance between CE toxicity reduction through sorption and substrate bioavailability, while Char700, Char900, and activated carbon sorbed CEs more strongly, were associated with less PCE dechlorination and lower ethene formation rates and favored methanogenesis. Among the tested materials, Char500 was particularly effective in our system, reviving *Dehalococcoides* rapidly, promoting the fastest PCE dechlorination, while minimizing methanogen stimulation. These results suggest that high-temperature biochars or activated carbons, which require greater energy inputs to produce, may not be necessary when intermediate-temperature biochar Char500 already achieves strong performance. Although the lower-pyrolysis-temperature chars, Char350 in particular, may be less stable and thus decompose more rapidly than biochars pyrolyzed at higher temperatures, they are still relatively recalcitrant black carbons^99,100^ that can offer several distinct advantages for bioremediation.

Correlations between physiochemical biochar properties and ethene production rates shed light on tuning material properties to enhance specific microbial activity and on designing sustainable materials beneficial for a wider range of bioprocesses that potentially enhance ethene or methane production. These findings could inform future applications of anaerobic complete PCE bioremediation strategies using poplar biochar and reveal tunable material properties that achieve desired enhancement of targeted biological activities (i.e., dechlorination/ethene production vs methanogenesis).

By integrating CE reduction and ethene production data with molecular microbial ecology measurements (including qPCR for biomarker genes and metatranscriptomics) from both attached and suspended cell fractions, we developed mechanistic hypotheses about microbial biofilm formation, corrinoid salvaging and transport, and cell resuscitation pathways that could play a role in *Dhc* revival within mixed communities. Although biochar clearly stimulated *Dhc* revival, enhanced *Dhc* growth, and allowed complete PCE dechlorination to ethene under stressed conditions, this study did not directly confirm the underlying mechanism. Future research should further explore mechanisms through targeted tests, such as varying initial PCE concentrations with and without biochar, direct measurements of corrinoids, hydrogen monitoring, and microscopy to visualize attached *Dhc* and other microbes colonizing biofilms on biochar.

Although experiments were performed in batch microcosms, the apparent balance between CE toxicity reduction through sorption and substrate accessibility has clear implications for choosing biochar for field applications. Future research should examine Char500-like materials in microcosms or flow-through column systems containing site material (i.e., contaminated groundwater and aquifer sediment) to incorporate site-specific geochemistry. This will help assess the transport, durability, and effectiveness of biochar under conditions more closely resembling actual in situ remediation.

## Supplementary Material



## Data Availability

Sequencing data are in the Sequence Read Archive (SRA) under BioProject PRJNA1054096.^43^

## Notes

Supporting methods/results for chemical analysis, biochar preparation/characterization, biochar adsorption/desorption isotherms, micropore size, ζ potential, and EDC titration curves, culturing conditions and controls, mass balances including sorption, ethene/methane production rates, active SDC-9 experiments with biochar and activated carbons, volatile fatty acid production and electron balance, nucleic acid extraction, purification, and quantitation, and qPCR, DNA/RNA-seq, MAG recovery, statistics, taxonomy classification and function annotation, metatranscriptomics analysis, and correlations analyses (PDF)
