# Supplementary material for: The Effects of Biochar on the Revival and Performance of an Organohalide-Respiring Mixed Culture
Source: Environ Sci Technol. 2026 Apr 3;60(14):10883–97. doi: 10.1021/acs.est.5c13638 (PMC13085523; doi:10.1021/acs.est.5c13638)
Supplement: Supplementary file 1 [file es5c13638_si_001.pdf]

Supplemental Information for:

The effects of biochar on revival and performance of an organohalide-respiring mixed culture.

Weilun Zhao[1a], Hongyu Dang[1a], Han Cao[2], Sumbul Hafeez[2], Wenqing Xu[2] and Timothy E. Mattes[1]*

[1]Department of Civil and Environmental Engineering, 4105 Seamans Center, The University of Iowa, Iowa City, IA, 52242, USA

[2]Department of Civil Engineering, 140 Tolentine Hall, Villanova University, Villanova, PA, 19085, USA

a: these authors contributed equally

*Corresponding author

Email address: tim-mattes@uiowa.edu

This Supplemental Information document includes:

42 pages (S1-S42)

Supplemental Methods (Section S1-S6)

20 Figures

10 Tables

Section S1: Biochar preparation and characterization methods

Particle Sizer and Zeta Potential Analyzer NanoBrook Omni (Brookhaven, USA) were used to measure the point of zero charge (PZC) of biochar using Phase Analysis Light Scattering (PALS) mode. All biochar samples (0.5 g L^{-1}) were adjusted to the pH range of 2–10 in DI water to obtain the PZC. The average of at least three measurements at 25 °C were recorded.

X-ray photoelectron spectroscopy (XPS) spectra were obtained from a PHI 5000 VersaProve with 200 µm, 50 W beam using 117 eV and 23 eV pass energies, respectively. Energy calibration was performed at 284.8 eV binding energy for C1s spectra on all XPS data.

Elemental analysis of poplar biochar was performed by Galbraith Laboratories (Knoxville, TN) with a Flash 2000 Elemental Analyzer. Energy dispersive spectroscopy (EDS) was performed on a Hitachi S-4800 scanning electron microscope (SEM, Tokyo, Japan) equipped with an EDS accessory using a working distance of 15 mm and accelerating voltage of 20 keV. All samples were homogeneously coated with a 6 nm Au layer to prevent the charging effect.

Biochar conductivity was measured using a cylindrical pack bed containing two copper pistons. Each biochar sample was packed into the bed, and the resistance was measured using a digital multimeter. The length and the diameter before and after packing biochar were measured using a vernier caliper. Conductivity (S/m) was calculated by the following equation (eq. 1):

$$\sigma = (l - l_0)/((R - R_0) * A) \qquad \textbf{eq. 1}$$

where l and l_0 (m) were the length of the unit before and after the sample was packed; R and R_0 (Ω) were the resistivity of the unit before and after the sample was packed; A (m^2) represents the area of the cylindrical copper.

The electron-donating capacities (EDC) of all char were determined using a potentiometric titration method (**Figure S5**) adapted from our previous study.[1] Briefly, 1g of char was mixed with 400 mL 0.2 M ammonium buffer (pH 10), followed by the addition of excess $NaBH_4$ (0.025 M) for complete reduction of the char sample. Then, the titration was conducted drop by drop to the char suspension using an oxidizing agent (0.05 M I_2 in ammonium buffer) under an argon atmosphere in the dark. During the titration, the redox potential [millivolts (mV) versus standard hydrogen electrode (SHE)] of the suspension was recorded against the volume of the titrant (**Figure S5**), which was converted to the EDC of the sample using the equation (**eq.2**), assuming an eight-electron oxidation and a two-electron reduction of $NaBH_4$ and I_2 occurred, respectively, at pH 10.

$$EDC\ (mmol_{e-}/g_{char}) = C_i \cdot V_i \cdot \frac{2e-}{m_c} \qquad \textbf{eq. 2}$$

where C_i represents the concentration of the titrant (I_2), V_i represents the volume of the titrant added, m_c represents the mass of char in the suspension, and $2e^-$ means the number of electrons accepted by each I_2 molecule, assuming that I^- is the final product. The EDC was calculated based on the I_2 consumption at 535 mV, where the excess $NaBH_4$ and the EDC of char were completely exhausted.

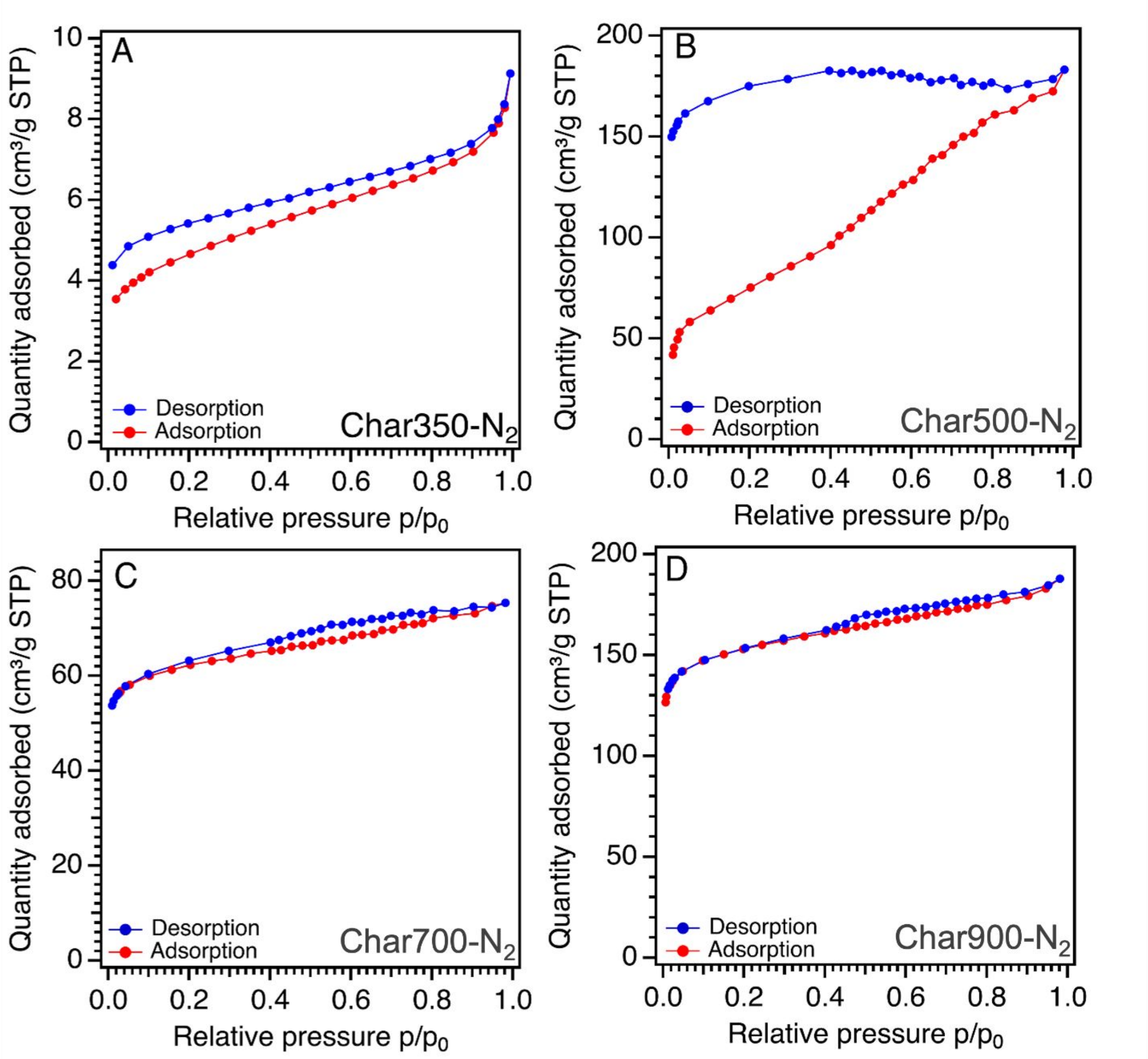

Figure S1. Nitrogen (N_2) adsorption and desorption isotherms of (A) Char350, (B) Char500, (C) Char700, and (D)Char900 at 77K. Before the measurements all samples were outgassed at 200°C for 16 h under vacuum. Because all Chars were produced at a temperature higher than 200°C, this outgassing temperature does not alter Char physicochemical properties.

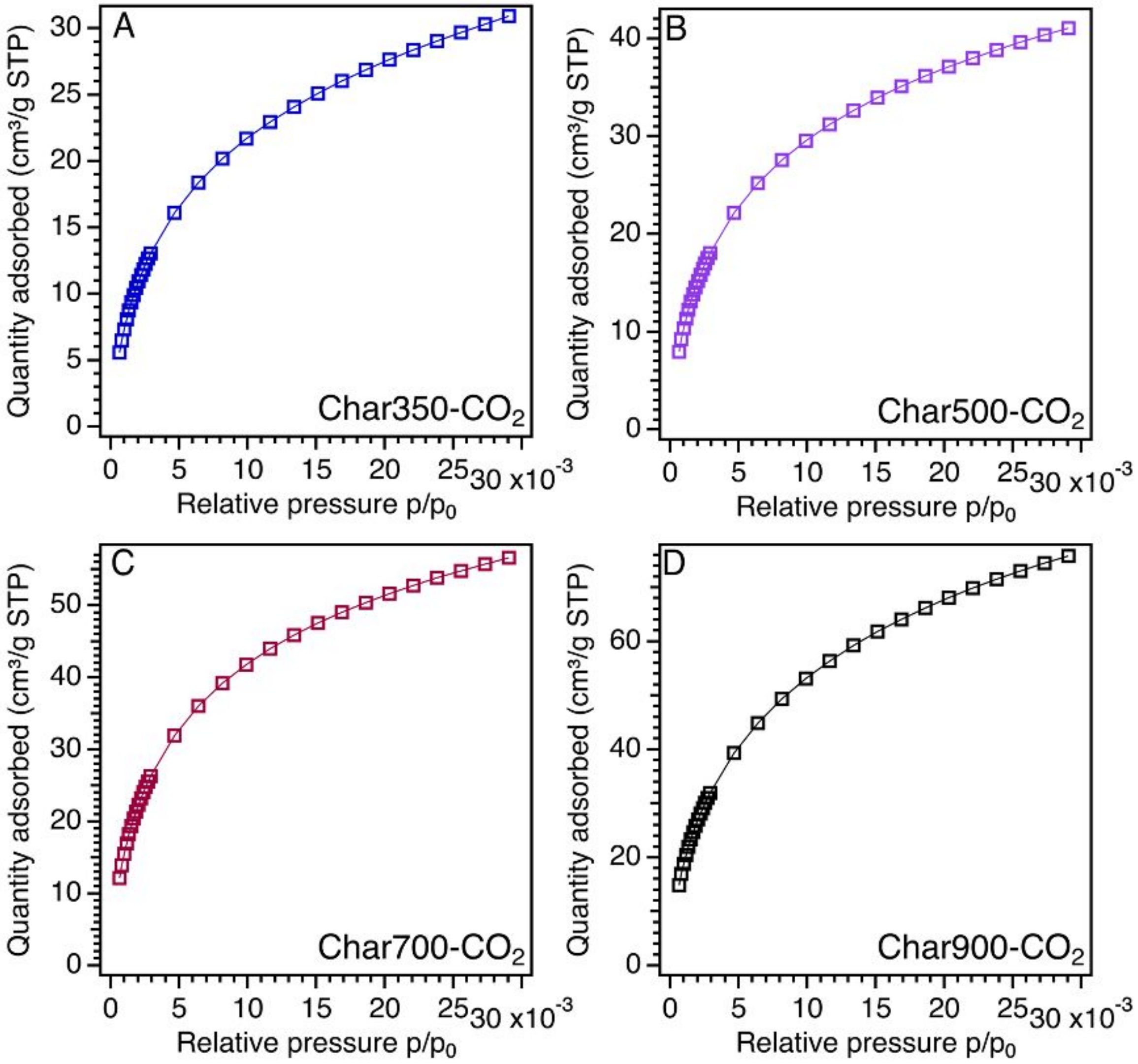

Figure S2. Carbon dioxide (CO_2) adsorption isotherm of (A)Char350, (B)Char500, (C)Char700, and (D)Char900 at 273K. Before the measurements all samples were outgassed at 200°C for 16 h under vacuum. Because all Chars were produced at a temperature higher than 200°C, this outgassing temperature does not alter Char physicochemical properties.

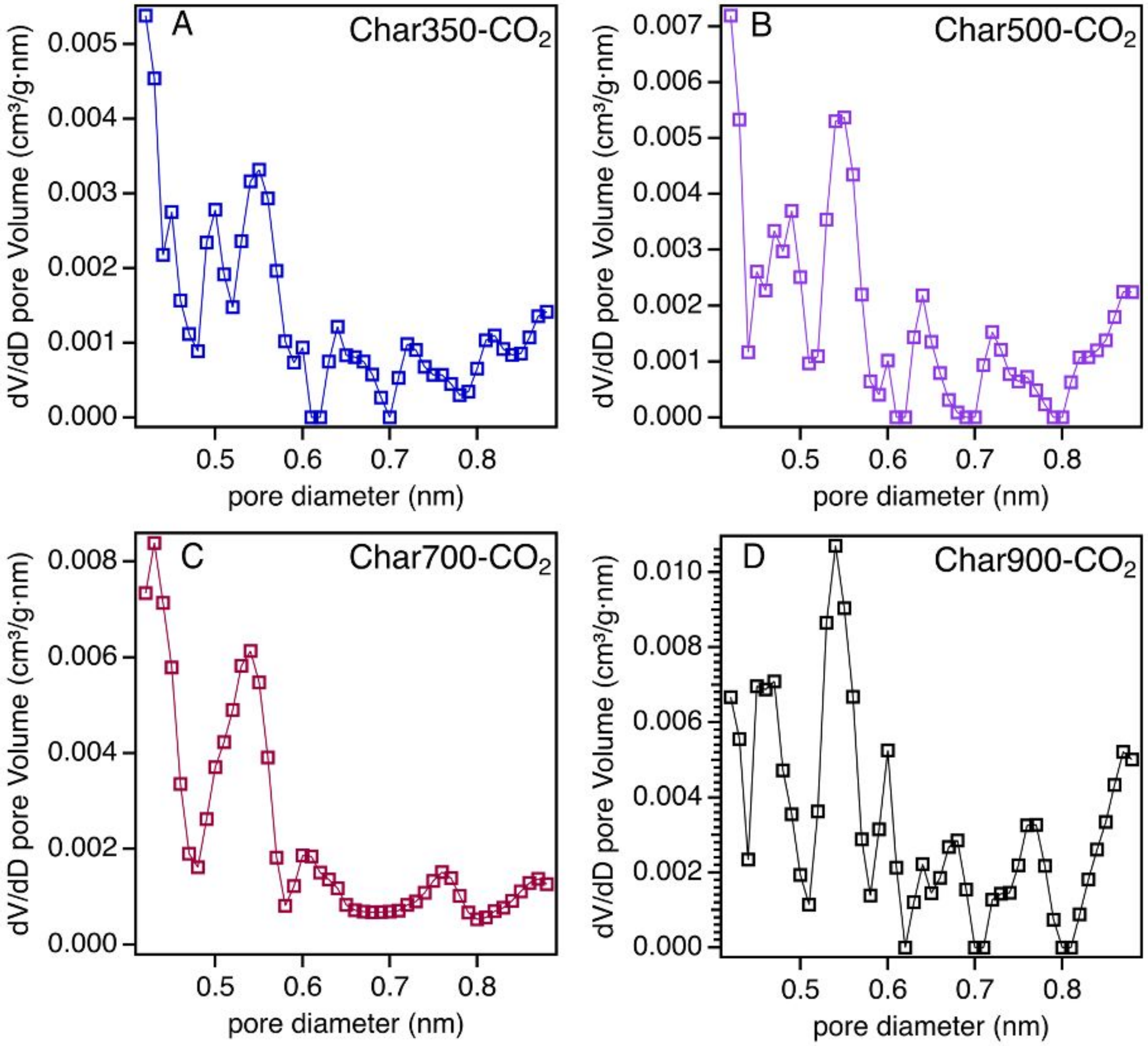

Figure S3. Micropore size distributions of (A)Char350, (B)Char500, (C)Char700, and (D)Char900 calculated from the grand canonical Monte Carlo (GCMC) model.

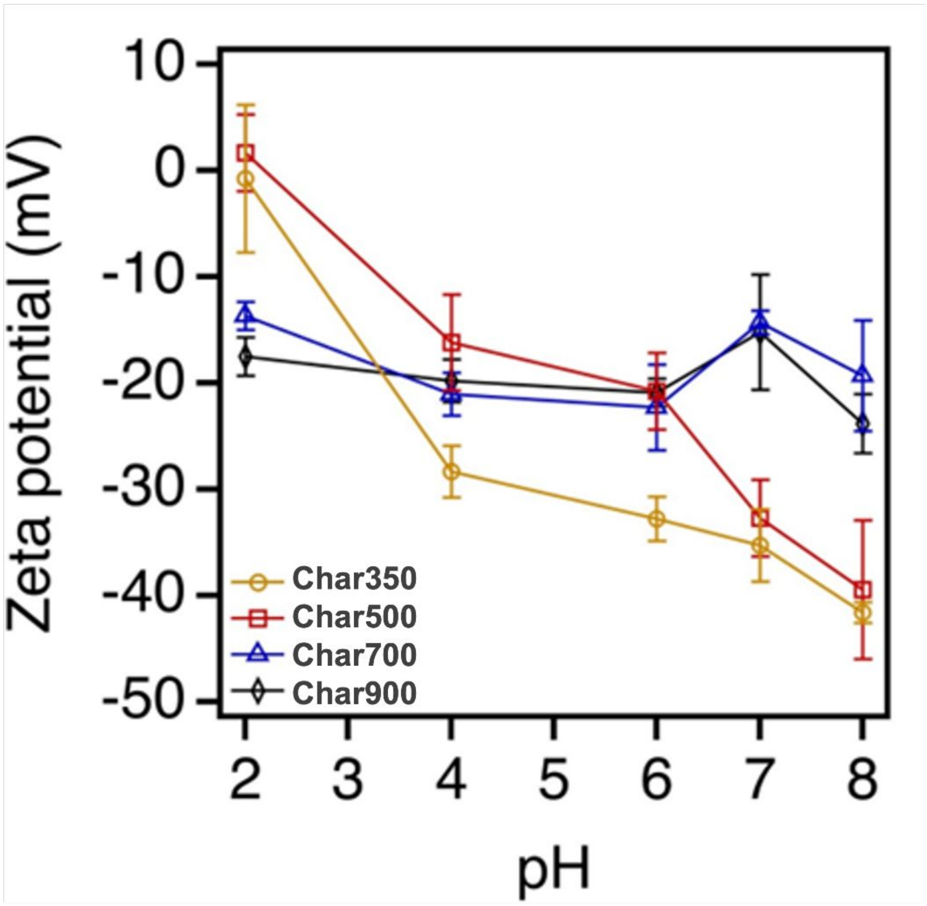

Figure S4. Zeta potential measurements of different biochar types (Char350, Char500, Char700 and Char900) with a solid-to-liquid ratio of 0.05 g/L in DI water.

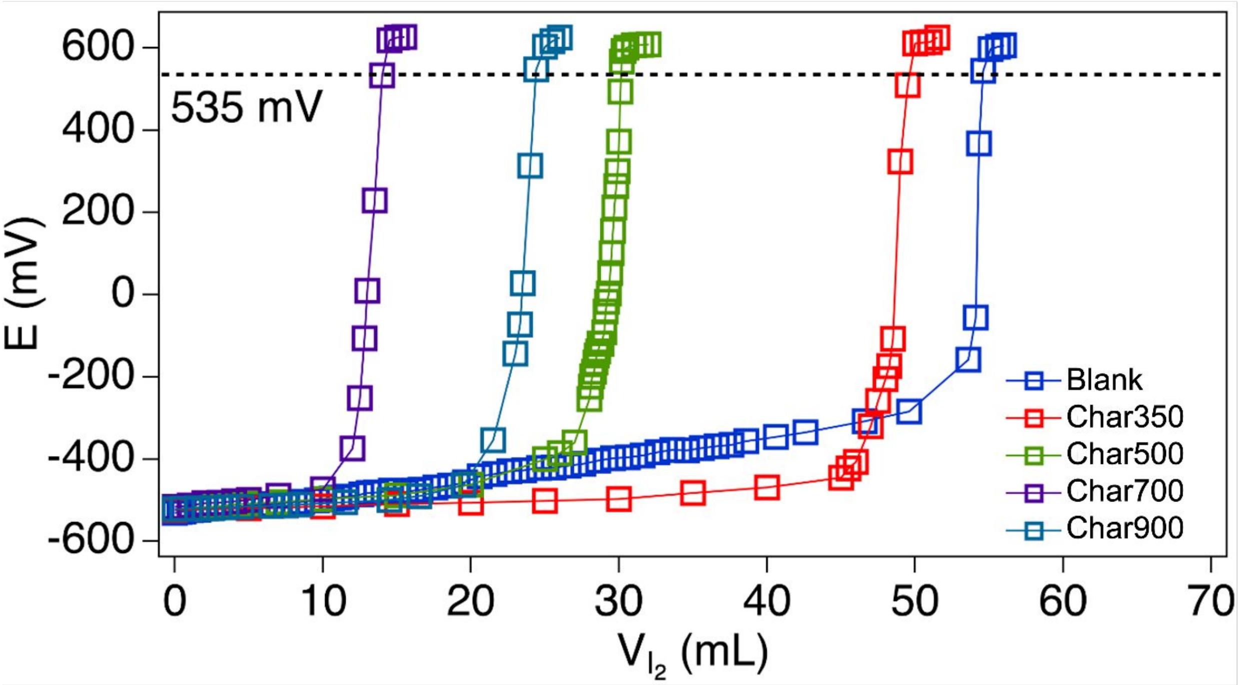

Figure S5. Titration curve for electron donating capacity of the blank (blue), Char350(red), Char500(green), Char700(purple), and Char900(cyan).

Section S2. Development of dormant and active dechlorinating SDC-9 inoculum used in experiments.

Dormant culture (10 ml) was inoculated into a bottle containing 90 ml RAMM, fed PCE (97.6 µmol) and lactate (1068 µmol) and incubated at 23°C with shaking (100 rpm) in the dark. Lactate (1068 µmol) was subsequently fed every four days, and chlorinated ethenes, ethene and methane were monitored by GC-FID for 55 days. PCE was reduced to cDCE after 20 days with some production of VC after 10 days, but no ethene (Figure S6). VC production indicated that Dhc was active and growing in this initial revived culture, so it was subsequently used as the inoculum for the experiment described in Figure 1.

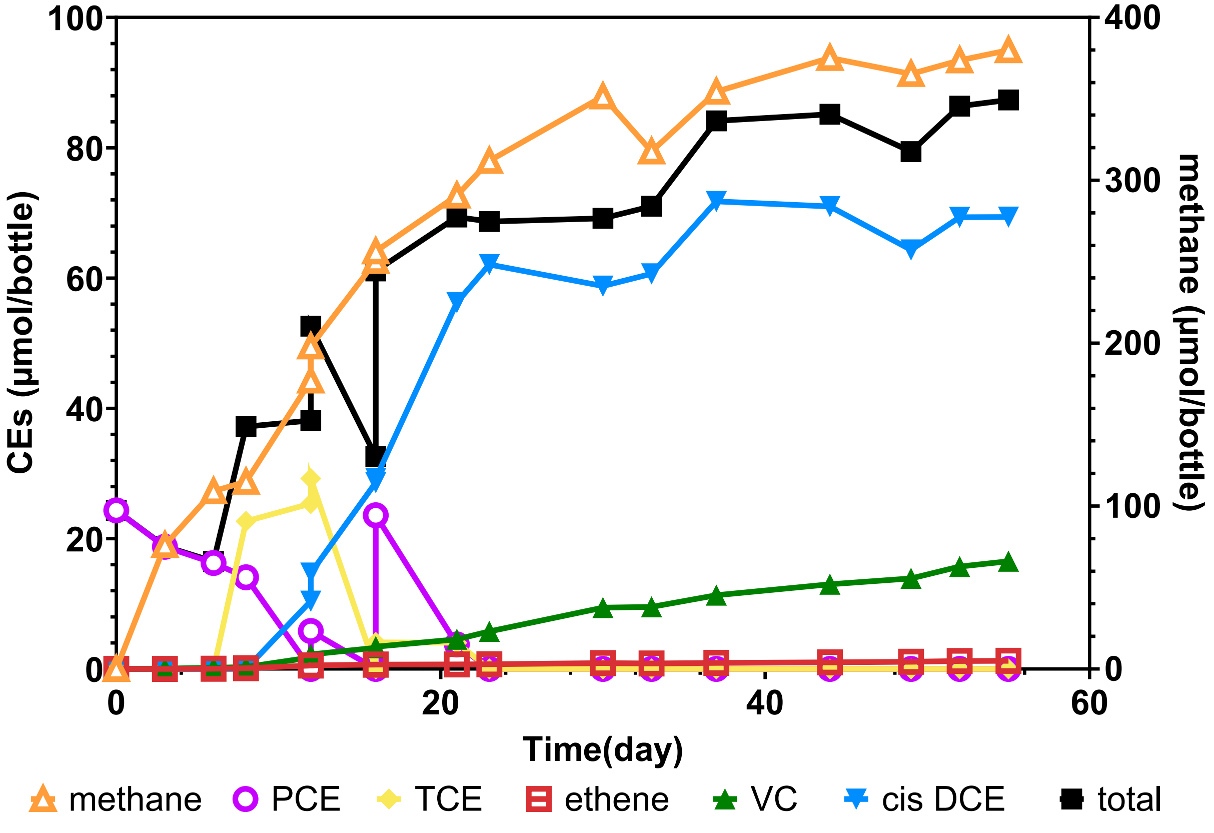

Figure S6. Changes in the mass per bottle (aqueous +gas phase) of chlorinated ethenes (PCE, TCE, cis-DCE, VC), ethene, and methane with time in a PCE-fed SDC-9 culture, which was developed from a dormant SDC-9 culture stored at 4°C for 2 years. This culture was used as inoculum in experimental bottles.

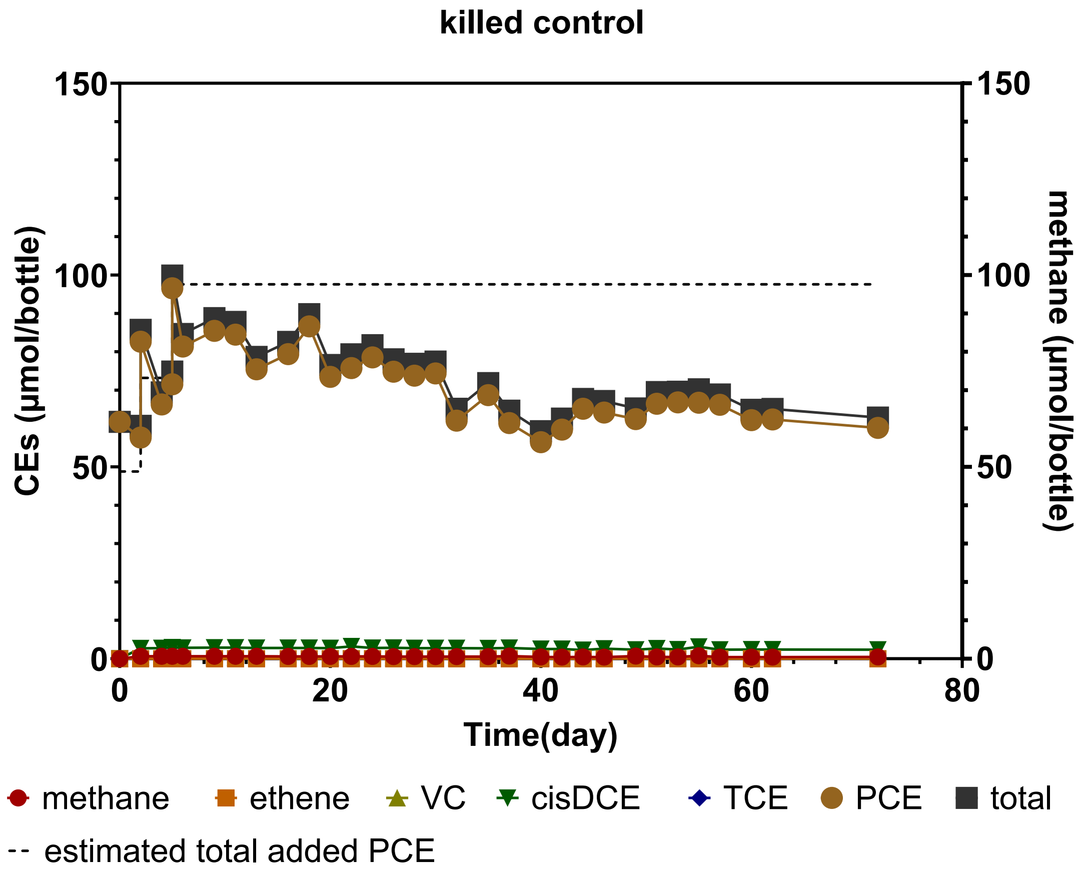

Figure S7. Changes in the mass of methane and chlorinated ethenes (PCE, TCE, cis-DCE, VC, and ethene) with time in killed SDC-9 cultures. Perchloric acid (770 µl) was added as the microbial poisoning agent to the killed control bottle prior to inoculation. There were no materials in this control bottle. This bottle was fed PCE and lactate in a similar manner to the live controls (no materials). No PCE transformation occurred (i.e., no dechlorination to TCE, cDCE, VC, or ethene). The change in PCE mass with time is attributed to sorption to bottle materials (butyl rubber septum).

Table S1. The retention time of methane, ethene, VC, *cis*-DCE, TCE, and PCE on GC-FID. The GC separation method begins at 90°C and holds 2 min to elute methane, ethene, and VC then the temperature ramps at a rate 35°C/min to 200°C and holds for 5 min to separate PCE, TCE, and cis-DCE.

Chemical	RT (min)
Methane	0.493
Ethene	0.631
VC	1.623
Cis-DCE	4.417
TCE	6.113
PCE	9.369

Table S2a. VC, ethene, and methane gas GC standard preparation.

Standard	Methane (ml)	Ethene (ml)	VC (ml)	Analyte methane mass (µmol/bottle)	Analyte ethene mass (µmol/bottle)	Analyte VC mass (µmol/bottle)
1	0.1	0.1	0.1	7.0	8.2	16.7
2	0.25	0.25	0.25	17.6	20.5	41.6
3	0.5	0.5	0.5	35.1	41.0	53.1
4	0.75	0.75	0.75	52.7	61.5	124.7
5	1	1	1	70.3	52.1	166.5

Table S2b. PCE, TCE, cDCE gas GC standard preparation. Mass of PCE, TCE, and cDCE added into 100 ml water per bottle.

Standard	PCE (µmol)	TCE (µmol)	cDCE (µmol)
1	34.2	30.0	26.5
2	20.5	17.8	15.9
3	13.7	12.2	10.6
4	6.8	6.1	5.3
5	1.5	1.7	2.0

Table S3. Sorption equilibrium data with Chars and CE+E – initial mass added, equilibrium aqueous phase concentrations (Ce), sorbed phase concentrations (Cs), and estimated partition coefficients (Kd). Sorption experiments were conducted in duplicate 160 mL bottles with 100 ml RAMM, biochar (0.2 g), and known initial masses of CEs and E. Bottles were incubated at 23°C with shaking (100 rpm) in the dark. CE and E concentrations (Ce) were measured by GC-FID after 1 month of equilibration. The solid phase concentrations (µmole/g) were determined using a mass balance approach ((Initial CE or E mass) – (aqueous and headspace CE or E mass) at equilibrium/g sorbent added (biochar)). Data shown are the average of duplicate measurements ± the standard deviation. Partition coefficients K_d (L/g) were calculated for ethene, VC, *cis*-DCE, TCE and PCE in equilibrium with each biochar with equation:

$$K_d = \frac{Concentration\ in\ the\ solid\ phase,\ Cs\ (\frac{umol}{g})}{Concentration\ in\ the\ liquid\ phase,\ Ce\ (\frac{umol}{L})}.$$

a. CE and E sorption data for Char 350 and Char500.

Compound	Initial mass (µmol)	Char350			Char500		
		Ce (µmole/L)	Cs (µmole/g)	K_d (L/g)	Ce (µmole/L)	Cs (µmole/g)	K_d (L/g)
PCE	50	120.5±30.7	166.3±21.4	**1.4±0.5**	65.1±5.5	204.8±3.8	**3.2±0.3**
TCE	25	90.6±7.2	70.2±4.3	**0.8±0.1**	22.3±0.9	111.5±0.6	**5.0±0.2**
cDCE	25	166.2±6.3	34.9±3.4	**0.2±0.0**	71.6±2.9	86.2±1.6	**1.2±0.1**
VC	25	142.8±7.7	10.4±6.2	**0.1±0.0**	110.2±3.7	36.5±2.9	**0.3±0.0**
ethene	25	43.9±0.9	7.6±2.3	**0.2±0.1**	42.7±0.6	10.8±1.6	**0.3±0.0**

b. CE and E sorption data for Char700 and Char900.

Compound	Initial mass (µmol)	Char700			Char900		
		Ce (µmole/L)	Cs (µmole/g)	K_d (L/g)	Ce (µmole/L)	Cs (µmole/g)	K_d (L/g)
PCE	75	84.5±20.8	316.2±14.5	**3.9±1.1**	10.3±1.6	367.9±1.1	**36±5.9**
TCE	50	11.6±0.7	243.0±0.4	**21±1.3**	8.0±0.9	245.1±0.6	**31±3.7**
cDCE	50	43.7±0.2	226.3±0.1	**5.2±0.0**	58.9±6.3	218.1±3.4	**3.7±0.5**
VC	25	69.1±2.6	69.6±2.1	**1.0±0.1**	88.6±6.2	53.8±5.0	**0.6±0.1**
ethene	25	43.9±4.3	8.0±11.3	**0.2±0.3**	36.8±2.8	26.6±7.4	**0.7±0.3**

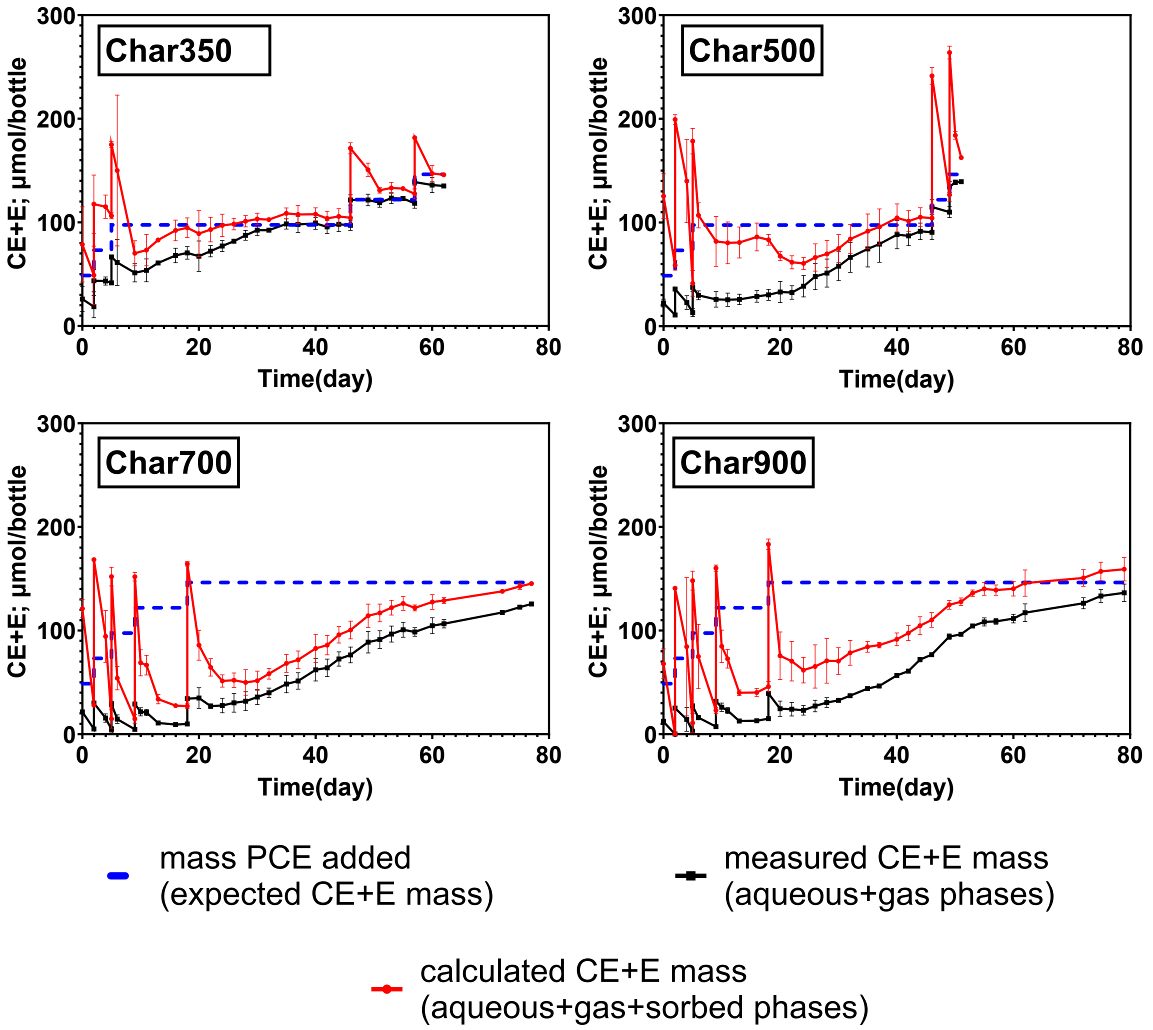

Figure S8. Comparison of chlorinated ethenes (CEs; PCE, TCE, cis-DCE, VC) + ethene (E) mass balances in PCE-fed SDC-9 cultures against the mass of PCE added per bottle (expected CE+E mass). The measured CE + E mass balance (measured aqueous + gas phases) was determined by GC measurements. The calculated CE+E mass balance (aqueous+ gas+calculated solid phase concentration (Cs)) was determined by estimating the sorbed CE+E mass on biochar using K_d values determined for each Char (data provided in Table S3). Because this K_d-based calculation assumes equilibrium, it is intended to show trends in sorption effects rather than exact mass closure at every sampling time.

Estimation of ethene and methane formation rates.

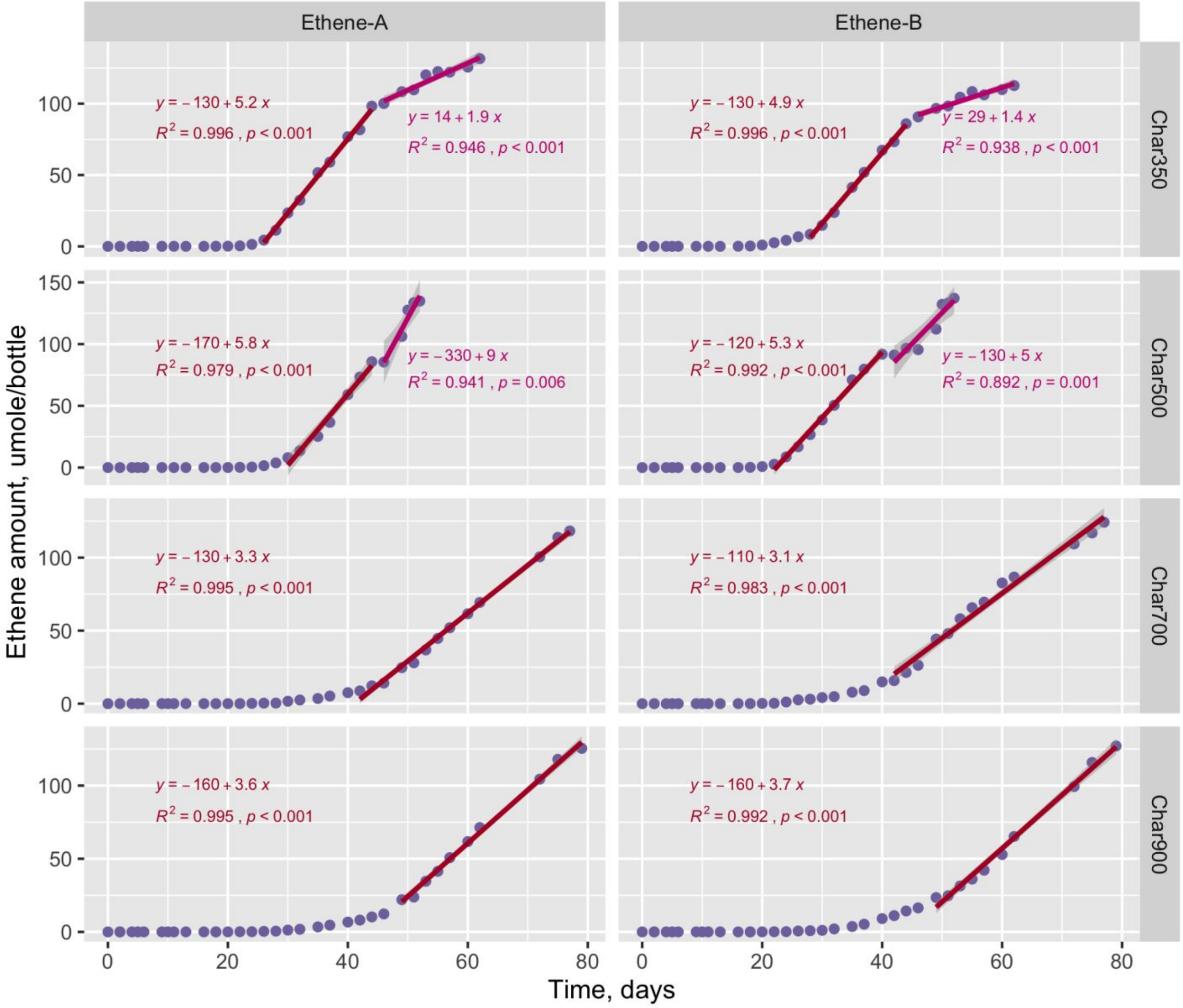

Figure S9. Estimation of ethene production rates in the Char350, Char500, Char700, and Char900 treatment bottles by applying a linear regression of ethene masses measured over a range of time points. Ethene-A and Ethene-B represent replicate bottles.

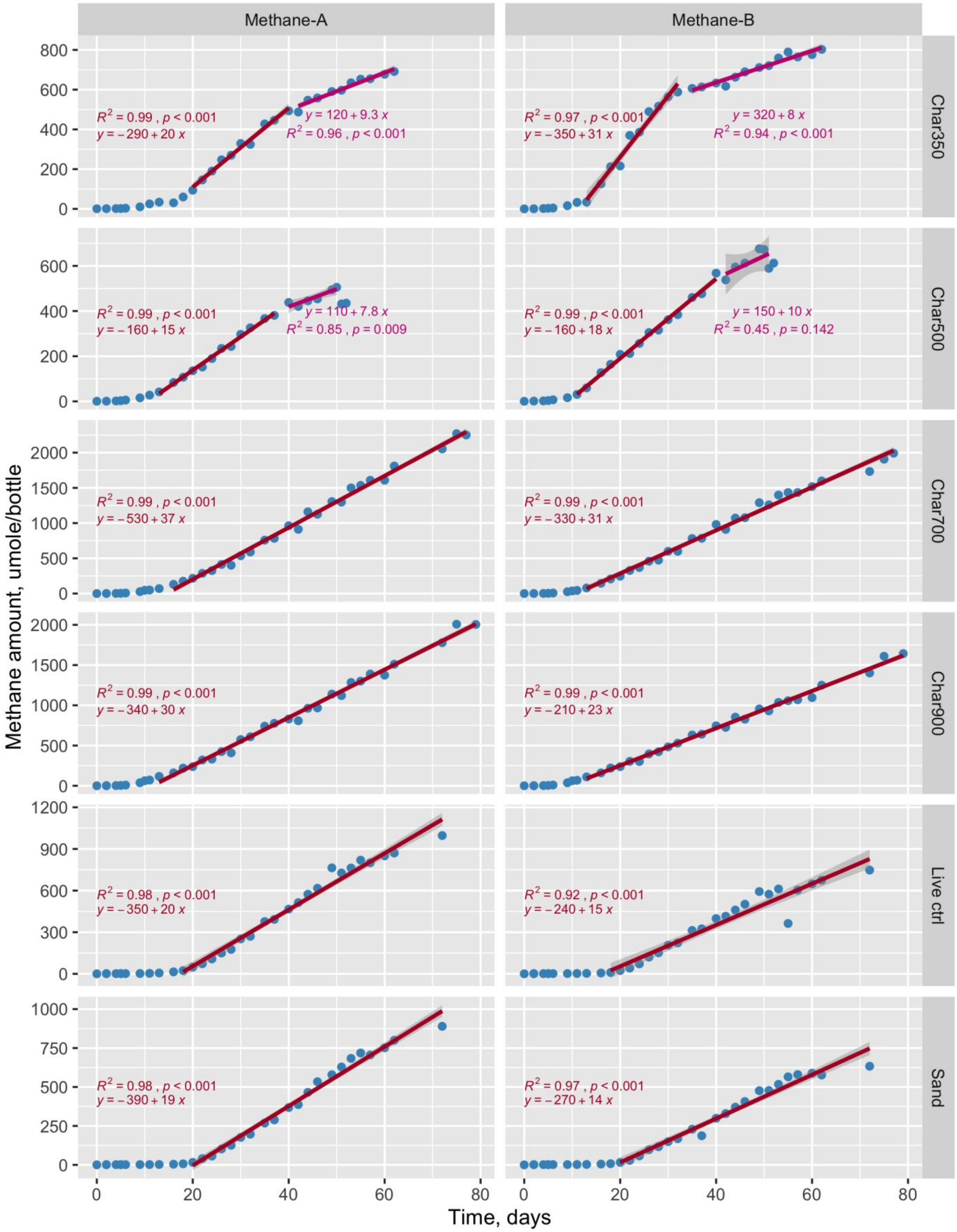

Figure S10. Estimation of methane production rates in the Char350, Char500, Char700, and Char900 treatment bottles by applying a linear regression of measured methane mass per bottle over a range of time points. Methane-A and Methane-B represent replicate bottles.

Table S4. Ethene and methane production rates in each bottle for experiments using both dormant and highly active SDC-9 cultures. Extra PCE spikes were added on day 46 to Char350 and Char500 treatments. The ethene and methane production rates from the dormant culture experiment shown are the average of duplicate estimates from Figures S9 and S10 ± the range (maximum and minimum values).

	Dormant SDC-9 culture		Highly active SDC-9 culture	
	**Ethene production rate (µmol/bottle/day)**	**Methane production rate (µmol/bottle/day)**	**Ethene production rate (µmol/bottle/day)**	**Methane production rate (µmol/bottle/day)**
Char350	5.1 ± 0.2[1] 1.7 ± 0.3[2]	25.5 ± 5.5[3] 8.7 ± 0.7[4]	5.2 ± 0.1	8.5 ± 1.6
Char500	5.6 ± 0.3[5] 7.0 ± 2[6]	16.5 ± 1.5[7] 8.9 ± 1.1[8]	6.8 ± 0.2	15.4 ± 0.8
Char700	3.2 ± 0.1	34 ± 3	4.6 ± 0.1	22.1 ± 2.7
Char900	3.7 ± 0.1	26.5 ± 3.5	5.3 ± 0.5	26.0 ± 1.7
No material	-	17.5 ± 2.5	2.6 ± 0.2	16.2 ± 1.3
Sand	-	16.5 ± 2.5	2.0 ± 0.2	21.7 ± 3.4
AC200	-	-	4.6 ± 0.2	29.1 ± 1.6
AC400	-	-	4.1 ± 0.2	30.5 ± 1.0

[1] Average ethene production rate between days 26-44 (bottle A) and between days 28-44 (bottle B).
[2] Average ethene production rate between days 46-62 in both bottles A and B.
[3] Average methane production rate between days 20-40 (bottle A) and between days 13-32 (bottle B).
[4] Average methane production rate between days 42-62 (bottle A) and between days 35- 62 (bottle B).
[5] Average ethene production rate between days 30-44 (bottle A) and between days 22- 40 (bottle B).
[6] Average ethene production rate between days 46-52 (bottle A) and between days 42-52 (bottle B).
[7] Average methane production rate between days 13-37 (bottle A) and between days 11- 40 (bottle B).
[8] Average methane production rate between days 20-40 (bottle A) and between days 42- 51 (bottle B).

Experiments with highly active SDC-9 cultures

Highly active SDC-9 cultures (Dhc>$1x10^{11}$ cells/ml) were obtained from APTIM (Lawrenceville, NJ). Each of the 17 serum bottles was inoculated with 6 ml of this culture. The treatments included duplicate bottles with Char350 (2 g/L; 150-250 μm), Char500 (2 g/L; 150-250 μm), Char700 (2 g/L; 150-250 μm), Char900 (2 g/L; 150-250 μm), and two different granular activated carbons (Filtrasorb200 (2 g/L) and Filtrasorb400 (2 g/L), Calgon Carbon). Duplicate live controls were also constructed without any added materials or with 2 g/L sand. There was one killed control bottle. Each bottle contained 94 ml of RAMM medium, supplemented with 1 ml of yeast extract (0.5 g/50 ml) and 0.01 ml of Vitamin B12 (25 mg/100 ml). The cultures were fed PCE (48.8 μmol) and lactate (7.12 mM) and incubated at 23°C with shaking at 100 rpm in the dark. Lactate (7.12 mM) was then replenished every four days, and chlorinated ethenes, ethene, and methane were monitored by GC-FID over 17 days. Additional PCE (48.8 μmol) was later added to each bottle based on the extent of PCE dechlorination.

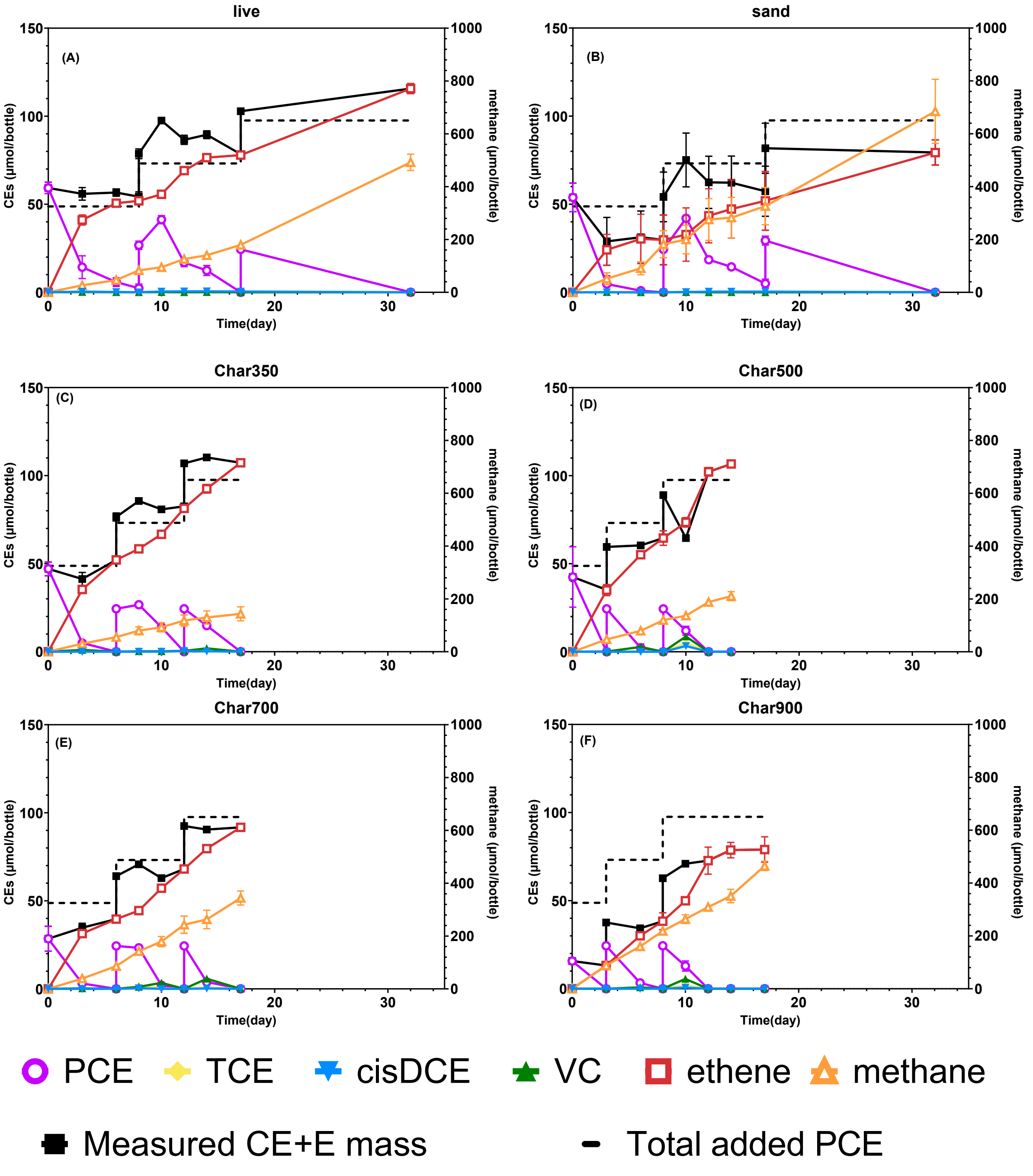

Figure S11. Changes in the mass per bottle (aqueous +gas phase) of chlorinated ethenes (PCE, TCE, cis-DCE, VC), ethene, and methane with time in actively dechlorinating PCE-fed SDC-9 culture. Dotted black lines show the estimated PCE mass spiked into bottles. The measured CE+E mass balance in each bottle is plotted (black squares). (A) SDC-9 without materials; (B) SDC-9 with sand; (C) SDC-9 with Char350; (D) SDC-9 with Char500; (E) SDC-9 with Char700; (F) SDC-9 with Char900. Data points are the average of duplicate bottles, and the error bars show the complete range of duplicates (maximum and minimum values). Data points that were below the detection of the instrument are shown as 0.

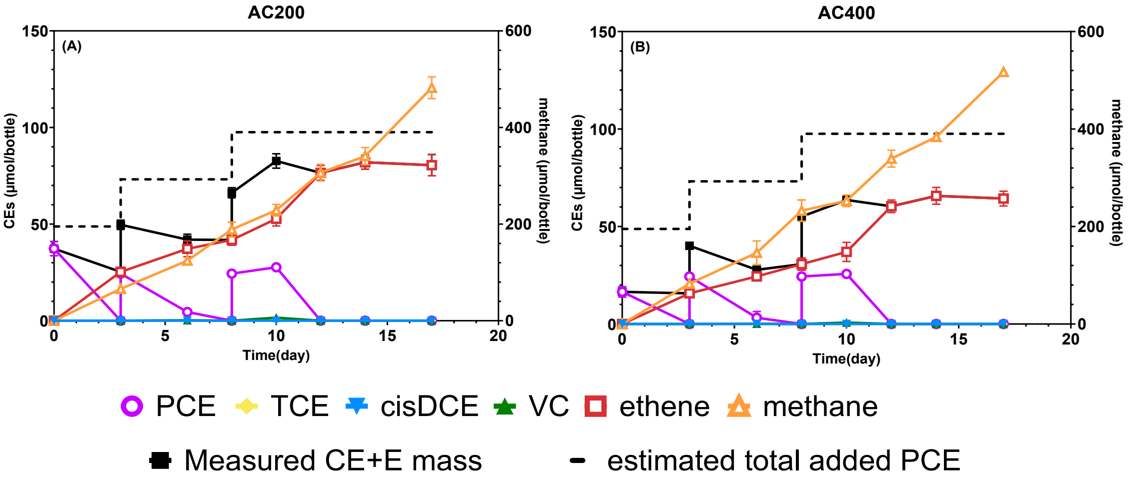

Figure S12. Changes in the mass per bottle (aqueous +gas phase) of chlorinated ethenes (PCE, TCE, cis-DCE, VC), ethene, and methane with time in actively dechlorinating PCE-fed SDC-9 culture. Dotted black lines show the estimated PCE mass spiked into bottles. The measured CE+ E mass balance in each bottle is plotted (black squares). (A) SDC-9 with granular activated carbon (Filtrasorb200, Calgon Carbon); (B) SDC-9 with granular activated carbon (Filtrasorb400, Calgon Carbon). Data points are the average of duplicate bottles, and the error bars show the complete range of duplicates (maximum and minimum values). Data points that were below the detection of the instrument are shown as 0.

Volatile fatty acid production and electron balance

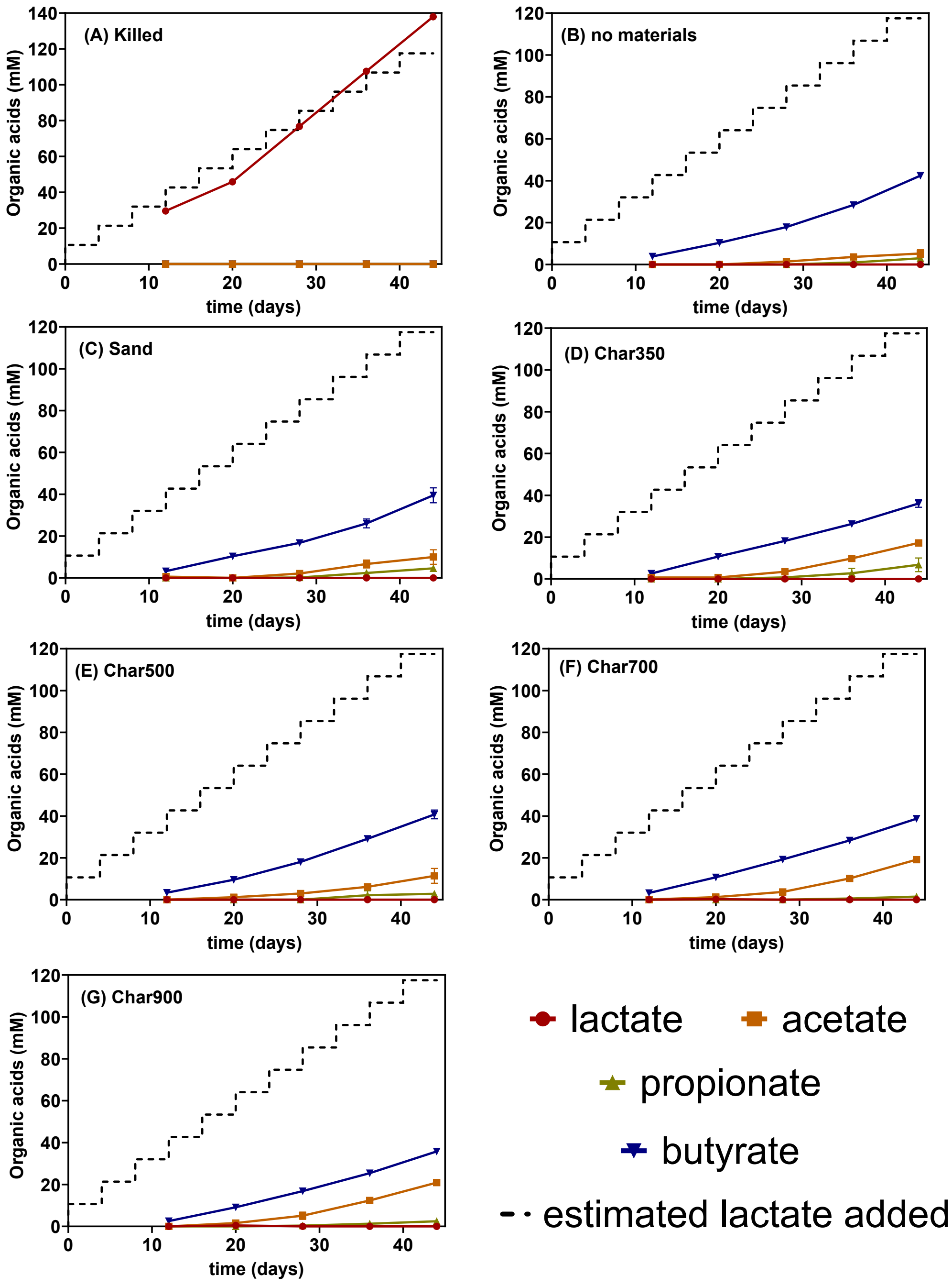

Figure S13. Concentration changes of organic acids with time. (A) killed SDC-9 control; (B) SDC-9 without materials; (C) SDC-9 with sand; (D) SDC-9 with Char350; (E) SDC-9 with Char500; (F) SDC-9 with Char700; (G) SDC-9 with Char900. Data points are the average of duplicate bottles (except the killed control, which is one bottle) and the error bars show the range of duplicates (maximum and minimum values).

Table S5. Proportion (%) of electrons (as eeq) originally from lactate in measured VFAs, CEs (TCE, cDCE, and VC), ethene, and methane in each treatment and control on day 46 and final sampling days. The number of eeq per mole of product is 12 (lactate), 8 (acetate), 14 (propionate), 20 (butyrate), 8 (methane), 8 (ethene), 6 (VC), 4 (cDCE) and 2 (TCE). The eeq values for CEs and ethene represent 2 eeq transferred onto PCE and dechlorination products during dechlorination to ethene. Unaccounted eeq in unmeasured products (e.g., biomass, hydrogen and other microbial processes) are grouped into the "other" category. Values are the average of two replicates ± the standard deviation.

**Product**	**Char350**		**Char500**	
	day 46	day 62	day 46	day 51
methane	3.5±0.5	2.9±0.3	3.0±0.6	2.7±0.6
ethene	0.6±0.0	0.5±0.1	0.5±0.0	0.7±0.0
VC	0.0	0.0	0.0	0.0
cDCE	0.0	0.0	0.0	0.0
TCE	0.0	0.0	0.0	0.0
acetate	9.8±0.8	-	6.5±2.8	-
propionate	6.7±4.6	-	2.8±2.1	-
butyrate	51.2±3.7	-	57.8±4.2	-
others	27.3±1.2	-	29.4±1.4	-
**Product**	**Char700**		**Char900**	
	day 46	day 77	day 46	day 79
methane	6.2±0.2	7.0±0.6	5.1±0.6	6.0±0.8
ethene	0.1±0.0	0.4±0.0	0.1±0.0	0.4±0.0
VC	0.2±0.0	0.0	0.2±0.0	0.0
cDCE	0.0	0.0	0.0	0.0
TCE	0.0	0.0	0.0	0.0
acetate	10.9±0.7	-	11.9±0.4	-
propionate	1.5±0.3	-	2.4±0.8	-
butyrate	55±1.6	-	50.8±2.5	-
others	26.1±0.7	-	29.5±1.9	-
**Product**	**sand**		**no materials**	
	day 46	day 72	day 46	day 72
methane	2.7±0.5	2.6±0.6	3.2±0.5	3.0±0.6
ethene	0.0	0.0	0.0	0.0
VC	0.0	0.0	0.0	0.0
cDCE	0.3±0.0	0.2±0.0	0.3±0.0	0.2±0.0
TCE	0.0	0.0	0.0	0.0
acetate	5.7±2.8	-	3.0±1.4	-
propionate	4.5±1.8	-	3.0±0.7	-
butyrate	56.0±7.2	-	60.2±2.5	-
others	32.3±2.0	-	31.3±1.3	-

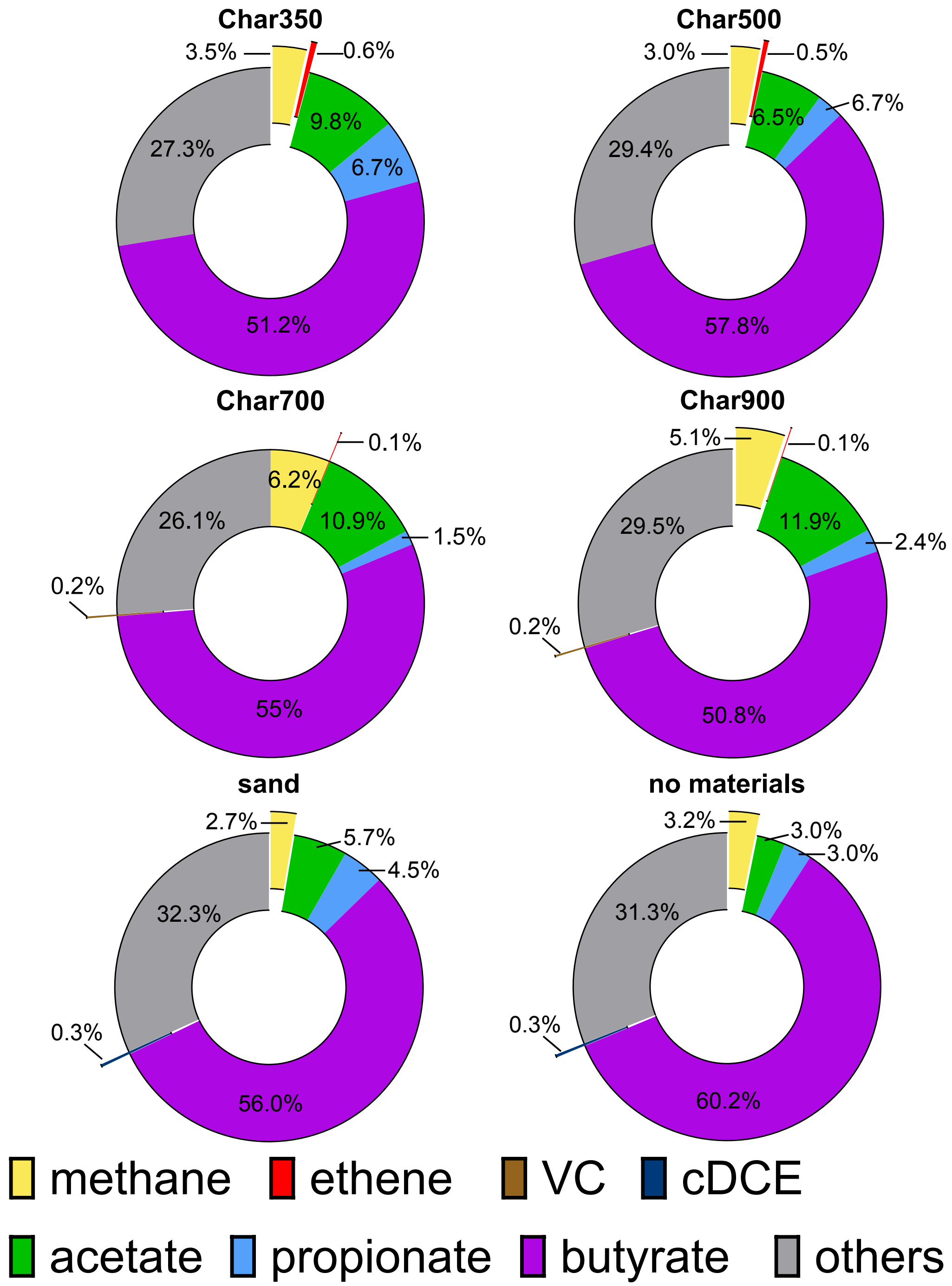

Figure S14. Proportion (%) of electrons (in eeq) originally from lactate in measured VFAs (acetate, propionate, butyrate), CEs (TCE, cDCE, and VC), ethene, and methane in each treatment and control on day 46, as detailed in Table S5. The "Other" category accounts for the remaining lactate eeq not detected in these pools, attributed to biomass formation and other microbial processes.

Section S3. Nucleic Acid Extraction, Purification, Abundance Quantitation with qPCR, and Quality Control

DNA was extracted from liquid culture samples (2 ml) using the DNeasy PowerWater Sterivex Kit (Qiagen, Hilden, Germany). Because Sterivex filters were not used, solution ST1B and the initial vortex step for releasing cells from filters were omitted from the protocol.[2] RNA was also extracted from liquid culture samples (13-14 ml) in all live controls and biochar treatments on the same days using the RNeasy PowerSoil Total RNA Kit (Qiagen). DNA and RNA from solid samples in biochar and sand bottles (200 mg dry mass) were extracted with the RNeasy PowerSoil Total RNA Kit (Qiagen) and the RNeasy PowerSoil DNA Elution Kit (Qiagen).

RNA extracts were treated with DNase using the TURBO DNA-Free Kit (Thermofisher Scientific, Waltham, MA), and then purified and concentrated with the Zymo RNA Clean and Concentrator Kit (Zymo Research Corporation, Irvine, CA) with additional on-column DNase treatment. RNA concentrations and purity were measured using a Nanodrop 2000 spectrophotometer (Thermo Scientific). Final RNA concentrations were measured with the Qubit RNA high sensitivity assay kit. RNA quality was further assessed with RNA Pico Assay on the Agilent 2100 Bioanalyzer (Agilent Technologies, Inc. Santa Clara, CA, USA).

DNA used for sequencing was further purified with RNase A (10 mg/mL, ThermoFisher Scientific) for 15 min at room temperature. DNA was cleaned and concentrated with the Zymo DNA Clean & Concentrator kit (Zymo Research Corporation). DNA concentrations and purity were measured using Nanodrop 2000 spectrophotometer (ThermoFisher Scientific). Final DNA concentrations were measured with the Qubit DNA high sensitivity assay kit. DNA quality was further analyzed on Agilent Genomic DNA ScreenTape System (Agilent Technologies, Inc. Santa Clara, CA, USA) prior to sequencing.

Table S6. Oligonucleotide primers and gBlock standard sequences for *Dehalococcoides* (*Dhc*) 16S rRNA, *vcrA*, *tceA*, *Dsf-pceA*, and *mcrA* target genes used for qPCR.

Target gene	Primer name	Sequences (5' – 3')	Product size (bp)	Reference
Dhc 16S rRNA gene	DHC-793F	GGGAGTATCGACCCTCTCTG	153	[3]
	DHC-946R	CGTTYCCCTTTCRGTTCACT		
vcrA	Vcr1022F	CGGGCGGATGCACTATTTT	71	[4]
	Vcr1093R	GAATAGTCCGTGCCCTTCCTC		
tceA	tceA-500F	TAATATATGCCGCCACGAATGG	295	[5]
	tceA-795R	AATCGTATACCAAGGCCCGAGG		
mcrA	mcrA_F3	CTTGAARMTCACTTCGGTGGWTC	271	[6]
	mcrA-rev	CGTTCATBGCGTAGTTVGGRTAGT		
Dsf-pceA	rdhA29_1488F	TCGAGACCTGGAACCACGAT	79	[7]
	rdhA29_1547R	TCATCAAACTTGCGGGCTG		
DHC_16S_*bvcA*_*vcrA* (gBlocks)		TGCATGATCTACGTGCGTCACATGCAGTACG<br>GGAGTATCGACCCTCTCTGTGCCGAAGCTAA<br>CGCTTTAAGTGTCCCGCCTGGGGAGTACGGT<br>CGCAAGGCTAAAACTCAAAGGAATTGACGG<br>GGGCCCGCACAAGCAGCGGAGCGTGTGGTTT<br>AATTCGATGCTACACGAAGAACTTACCAAGA<br>TTTGACATGCATGAAGTAGTGAACCGAAAGG<br>GTTTTTTGAATAGTCCGTGCCCTTCCTCACCA<br>CTACCAGGAAATGGTTGAGTTACTGCGTAAA<br>ATAGTGCATCCGCCCGTTTTTTAAAAGCACTT<br>GGCTATCAAGGACTTGGTGGCGACGTGGCTA<br>TGTGGGGACCTGGTGGTGCTTTTGGCACTAG<br>CTCAGATTCAGTAGACCGCTGTTG	397	This study

tceA (gBlocks)	TAATATATGCCGCCACGAATGGCTCACATAA<br>TTGCTGGGAGAACCCGCTTTATGGACGCTAT<br>GAAGGTTCTAGGCCTTATCTCTCTATGCGAA<br>CCATGAATGGAATAAACGGCTTGCATGAATT<br>TGGTCACGCAGATATCAAAACCACCAACTAC<br>CCGAAGTGGGAGGGTACGCCTGAAGAGAAC<br>CTGTTAATCATGCGCACCGCCGCGCGCTACT<br>TCGGGGCTTCTTCCGTTGGCGCCATTAAGAT<br>AACGGATAACGTGAAGAAAATCTTCTATGCC<br>AAAGCCCAGCCCTTTTGCCTCGGGCCTTGGT<br>ATACGATTA	318	This study
mcrA (gBlocks)	TGCATGATCTACGTGCGTCACATGCAGTACC<br>TTGAAACTCAATTCGGTGGATCCCAAAGGGC<br>TGCTGTTGTTGCAGCTGCTGCAGGTATTTCAA<br>CTGCATTTGCTACTGGAAATGCTCAAACTGG<br>TTTAAGTGCATGGTACTTATCTCAATACTTAC<br>ACAAAGAACAACATTCCAGATTAGGTTTCTA<br>TGGTTACGATTTACAAGATCAATGTGGTGCT<br>GCTAACACATTCTCCTTCAGAAATGATGAAG<br>GTTTACCTCTTGAAATGAGAGGACCTAACTA<br>CCCTAACTACGCAATGAACGCACTAGCTCAG<br>ATTCAGTAGACCGCTGTTG	331	Designed in this study based on [6]
Dsf-pceA (gBlocks)	TGCATGATCTACGTGCGTCACATGCAGTACT<br>CGAGACCTGGAACCACGATGTGGCCAGAATA<br>GCCACCCAAATACCATTGCTTCAGGATGCAG<br>CCCGCAAGTTTGATGACACTAGCTCAGATTC<br>AGTAGACCGCTGTTG	139	Designed in this study based on SDC-9 metagenome

Definition of each biomarker gene used in this study

Under anaerobic conditions, OHRB, particularly *Dehalococcoides mccartyi*, perform step-by-step process of reductive dechlorination on CEs such as PCE, converting them to TCE, then to less chlorinated intermediates cDCE, followed by VC, and finally to ethene.[8,9] These processes rely on reductive dehalogenase (RDase) enzymes encoded by specific functional genes. The *Dsf-pceA* gene codes for RDases that dechlorinate PCE into cDCE, *tceA* mainly reduces TCE and cDCE (with some activity toward VC), and *vcrA* catalyzes the reduction of cDCE and VC into ethene.[7,10-12] These genes, along with the *Dehalococcoides* 16S rRNA gene, serve as molecular biomarkers for evaluating the presence and activity of dechlorinating microbial populations. Methanogenic archaea are essential members of OHRB microbial communities. It is suggested that electrons transferred during methanogenesis are diverted to PCE by a reduced electron carrier involved in methane production, which is necessary for RDase activity.[13] The *mcrA* gene, encoding the alpha subunit of methyl-coenzyme M reductase, is commonly used as a biomarker for methanogens, allowing the assessment of their abundance and activity.

Dhc 16S: Partial 16S rRNA gene specific to the Genus *Dehalococcoides*. Used to estimate the abundance of *Dehalococcoides* in mixed cultures and environmental samples.

vcrA: The *vcrA* gene encoding VC reductive dehalogenase, which was able to dechlorinate cDCE and VC to ethene, and 1,2-dichloroethane to ethene, was isolated and characterized from *Dehalococcoides mccartyi* strain VS.[11,12]

tceA: The *tceA* gene that encodes the TCE reductive dehalogenase, which was found to participate in VC dechlorination but is more active in TCE and cDCE reduction, was found in *Dehalococcoides ethenogenes* strain 195.[10]

Dsf-pceA: The *Dsf-pceA* gene encodes the PCE reductive dehalogenase, which dechlorinates PCE and TCE to cDCE. The primer[7] and gBlock were designed for *Dehalobacter* in SDC-9 consortium genomic sequence.

mcrA: The *mcrA* gene encoding the alpha subunit of methyl-coenzyme M reductase was used to detect and quantify the abundance of methanogens. The primer was designed to target all methanogens, thus qPCR cannot differentiate between hydrogenotrophic and acetoclastic methanogen types.[6] The gBlock was designed according to the *Methanobrevibacter mcrA* sequence.

Gene copies/ml of liquid culture and gene copies/g of solid in qPCR were calculated using the following equations. Each bottle contained 0.2 g of solid, which was entirely collected for DNA extraction. Additionally, each bottle had 100 ml of liquid, from which 2 ml was taken for DNA extraction. The final DNA extraction volume was 100 µl per bottle, and 2 µl of this extract was used for qPCR in each well.

$$\frac{copies}{g} = \frac{copies}{well} * \frac{100\ \mu l\ total\ DNA\ extract}{\frac{2\ \mu l\ DNA\ extract}{well}} * \frac{1}{0.2\ g\ solid}$$

$$\frac{copies}{ml} = \frac{copies}{well} * \frac{100\ \mu l\ total\ DNA\ extract}{\frac{2\ \mu l\ DNA\ extract}{well}} * \frac{1}{2\ ml\ liquid\ sample}$$

$$\frac{copies}{bottle}(attached\ biomass) = \frac{copies}{well} * \frac{100\ \mu l\ total\ DNA\ extract}{\frac{2\ \mu l\ DNA\ extract}{well}}$$

$$\frac{copies}{bottle}(suspended\ biomass) = \frac{copies}{well} * \frac{100\ \mu l\ total\ DNA\ extract}{\frac{2\ \mu l\ DNA\ extract}{well}} * \frac{1}{2\ ml\ liquid\ sample} * 100\ ml\ liquid$$

Table S7. Pertinent qPCR parameters in accordance with MIQE guidelines.

Target gene	Primer concentration (μM)	qPCR linear range (gene copies/reaction)	qPCR efficiency	Y-intercept
Dehalococcoides 16S rRNA gene (qPCR)	50	$30 - 3 \times 10^7$	91.944	36.891
tceA (qPCR)	50	$30 - 3 \times 10^7$	97.156	34.542
vcrA (qPCR)	50	$30 - 3 \times 10^7$	99.177	35.101
mcrA (qPCR)	100	$30 - 3 \times 10^7$	80.123	41.657
Dsf-pceA (qPCR)	100	$26\text{-}2.63 \times 10^7$	101.22	32.445

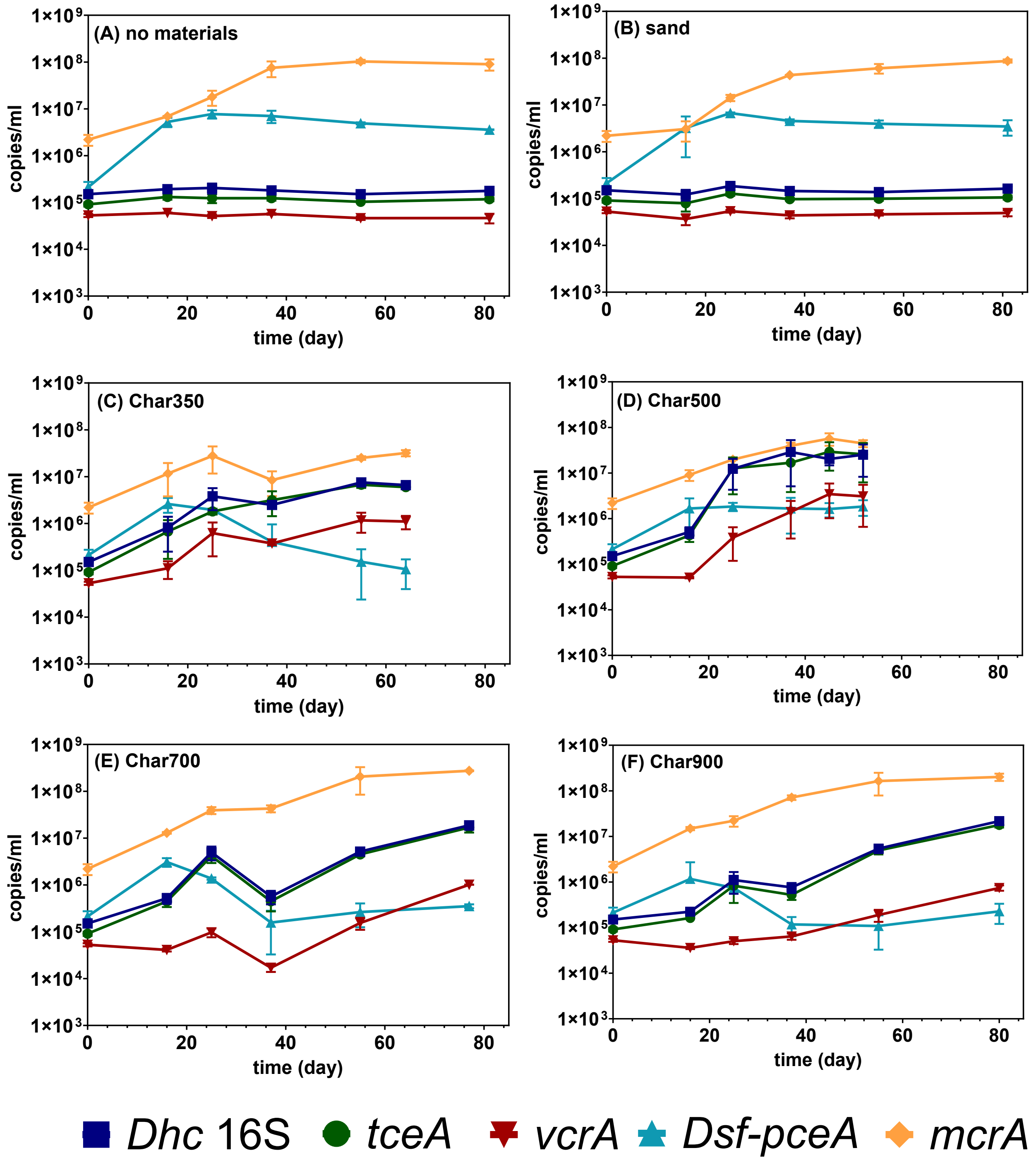

Figure S15. Abundance of CE dehalogenation (*Dhc* 16S, *vcrA*, *tceA, Dsf-pceA*) and methanogenesis *(mcrA*) biomarkers in liquid culture samples from (A) live control without materials; (B) live control with sand (2 g/L); (C) Char350 (2 g/L); (D) Char500 (2 g/L); (E) Char700 (2 g/L); (F) Char900 (2 g/L). The lower qPCR quantification limit is 1500 copies/ml. Data points represent the average of duplicate bottles, and the error bars are the complete range of duplicates (maximum and minimum values). Gene copies/ml of liquid sample calculations are described in SI Section S3.

Section S4. DNA and RNA sequencing.

DNA and RNA extracted from liquid samples and biochar samples were sequenced at the University of Iowa Institute of Human Genomics (IIHG; Iowa City, IA, USA). The Illumina TruSeq Stranded Total RNA Library Prep kit and Illumina Ribo-Zero plus rRNA Depletion Kit were used to prepare RNA libraries. Supplemental probes for rRNA depletion were developed by obtaining full 16S rRNA sequences of the 8 most abundant taxa in SDC-9 from the SILVA database (Table S8) and sending these sequences to Illumina (San Diego, CA) for probe development. Probes designed by Illumina were ordered from Integrated DNA Technologies (IDT; Coralville, IA). RNA libraries were pooled and sequenced on one lane of an S1 Illumina Novaseq 6000 (2×100 bp paired end reads) flow cell. The Roche KAPA HyperPrep Kit was used to prepare DNA libraries. DNA libraries were pooled and sequenced on separate lanes of an S Prime Illumina Novaseq 6000 (2×150 bp paired-end reads) flow cell.

Table S8. The 16S rRNA sequences from Silva database (release 138.1) used to design probes for rRNA depletion during RNA library preparation.

Taxonomy	Silva database accession number
Eubacterium	QTVG01000004
Dehalococcides mccartyi	CP011127
Desulfitobacterium hafniense	AP008230
Desulfovibrio biadhensis	LM999902
Methanocorpusculum aggregans	LMVO01000026
Endomicrobium proavitum	CP009498
Macellibacteroides fermentans	HQ020488

DNA and RNA samples names, Sequence Read Archive (SRA) accession numbers, and NCBI BioSample numbers are listed in Table S9.

Table S9. Sample names, SRA accession numbers, and BioSample numbers of the samples subjected to DNA-Seq and RNA-seq in this study.

Sample name	SRA accession no.	BioSample no.
Char350-A-liquid_DNA	SRR27243496	SAMN38883632
Char350-B-attached_DNA	SRR27243495	SAMN38883633
Char500-A-attached_DNA	SRR27243484	SAMN38883634
Char500-B-liquid_DNA	SRR27243476	SAMN38883635
Char700-A-attached_DNA	SRR27243475	SAMN38883636
Char700-B-liquid_DNA	SRR27243474	SAMN38883637
Char900-A-liquid_DNA	SRR27243473	SAMN38883638
Char900-B-attached_DNA	SRR27243472	SAMN38883639
NoChar-A-liquid_DNA	SRR27243471	SAMN38883640
Char350-A-attached_RNA	SRR27243470	SAMN38883641
Char350-A-liquid_RNA	SRR27243494	SAMN38883642
Char350-B-attached_RNA	SRR27243493	SAMN38883643
Char350-B-liquid_RNA	SRR27243492	SAMN38883644
Char500-A-attached_RNA	SRR27243491	SAMN38883645
Char500-A-liquid_RNA	SRR27243490	SAMN38883646
Char500-B-attached_RNA	SRR27243489	SAMN38883647
Char500-B-liquid_RNA	SRR27243488	SAMN38883648
Char700-A-attached_RNA	SRR27243487	SAMN38883649
Char700-A-liquid_RNA	SRR27243486	SAMN38883650
Char700-B-attached_RNA	SRR27243485	SAMN38883651
Char700-B-liquid_RNA	SRR27243483	SAMN38883652
Char900-A- attached_RNA	SRR27243482	SAMN38883653
Char900-A-liquid_RNA	SRR27243481	SAMN38883654
Char900-B-attached_RNA	SRR27243480	SAMN38883655
Char900-B-liquid_RNA	SRR27243479	SAMN38883656
NoChar-A-liquid_RNA	SRR27243478	SAMN38883657
NoChar-B-liquid_RNA	SRR27243477	SAMN38883658

Section S5. MAG recovery, taxonomy classification and function annotation

Trimmomatic (version 0.39)[14] was used to trim the raw sequencing reads. Megahit (version 1.2.9)[15] was then used to assemble the trimmed reads from DNA-seq into contigs (longer contiguous DNA segments) using a k-mer step of 10 and a maximum k-mer size of 127 with a combination of individual assembly and co-assembly approaches. The contigs were annotated with Prokka (version 1.14.6)[16] using *Dehalococcoides* genomes from NCBI as the reference database to provide an overview of the reductive dehalogenase (RDase) genes that were present. The contigs were also annotated with Prokka with the default database to provide an overview of methyl-coenzyme M reductase (*mcrA*) genes that were present. Short reads in DNA-seq samples were mapped to correlated contigs files with Bowtie2 (version 2.2.5).[17] Contigs longer than 1500 bases were binned with Metabat2 (version 2.15).[18] The contigs containing annotated reductive dehalogenase and *mcrA* genes were traced in the bins. Two contigs (NCBI accession: PP061217 and PP061218) containing RDase genes were not binned. One of the unbinned contigs (NCBI accession PP061217; 175252 bp) which contained the *vcrA* gene was designated as "vcrA_contig". Another unbinned contig (NCBI accession PP061218; 8598 bp) contained a *Dsf-pceA* gene and was designated as "*Dsf-pceA*_contig".

CheckM (version 1.2.2)[19] and Anvi'o (version 7.1)[20] were used to quantify the completeness and contamination/redundancy of the bins. Anvi'o was also used to refine the bins with a completion ≥80% and a contamination/redundancy >5%. Finally, a bin with a completion ≥80% and a contamination/redundancy ≤ 5% were designated as a metagenome assembled genome (MAG). dRep (version 3.4.2)[21] was used to dereplicate the final MAGs using 95% and 99% average nucleotide identity (ANI) for the primary and secondary clustering.

MAG taxonomy was determined using GTDB-Tk (version 2.1.1)[22] with database release R214. GTDB-Tk dependencies included: Prodigal (version 2.6.3),[23] HMMER (version 3.1b2),[24] pplacer (version v1.1.alpha19),[25] FastANI (version 1.3),[26] FastTree (version 2.1.11).[27]

MAGs were deposited into NCBI under Bioproject PRJNA1054096 and annotated with the NCBI Prokaryotic Genome Annotation Pipeline (NCBI-PGAP).[28] MAG coding sequences (CDS) identified by NCBI-PGAP were assigned KEGG Orthologs (KOs) by KofamScan.[29] MAGs were also annotated with Prokka (version 1.14.6)[16] to identify the rRNA and tRNA genes.

Reductive dehalogenase and methyl coenzyme M reductase (*mcrA*) genes were further verified by running an hmmsearch with HMMER (version 3.3.2)[24] against the HMM model for PF13486 (reductive dehalogenase domain), and PF02249 and PF02745 (methyl-coenzyme M reductase alpha subunit domain) downloaded from Pfam.[30] Annotated genes containing the key word "reductive dehalogenase" were further filtered to exclude genes annotated as "reductive dehalogenase membrane-anchoring subunit RdhB" or with an inferred protein sequence length of less than 400 amino acids from further analysis.

Table S10. MAG and contig statistics relevant to chlorinated ethene dehalogenation and methanogenesis in SDC-9 cultures. NA: not applicable.

MAG[a]/contig	Genbank accession	Genome size (bp)	# of contigs	Completion (%)	# of RDase genes	MAG Biosample ID	Biosamples used for read recruitment and assembly
MAGs and contigs with RDase genes							
Dehalococcoides mccartyi	GCA_034927175.1	1240863	4	91.09	5	SAMN38932800	Coassembly[b]
vcrA_contig	PP061217	175252	1	0.00	1	NA	Coassembly[b]
Desulfitobacterium	GCA_034927245.1	3358126	86	100.00	1	SAMN38932797	Coassembly[b]
Desulfitobacterium hafniense	GCA_034933185.1	5177525	66	98.98	2	SAMN38932734	SAMN38883634 (Char500-attached)
*Dsf-pceA*_contig	PP061218	8598	1	0.00	1	NA	Coassembly[b]
Petrimonas sp002356435	GCA_034933105.1	2537347	255	83.10	1	SAMN38932736	SAMN38883634 (Char500-attached)
Methanogen MAGs							
Methanocorpusculum parvum	GCA_034933645.1	1657343	21	99.54	0	SAMN38932711	SAMN38883634 (Char350-attached)
Methanocorpusculum	GCA_034932965.1	1555513	102	90.96	0	SAMN38932741	SAMN38883635 (Char500-suspended)
VadinCA11	GCA_034928305.1	1335961	6	100.00	0	SAMN38932760	SAMN38883638 (Char900-suspended)
Methanobrevibacter	GCA_034928265.1	2359402	41	100.00	0	SAMN38932764	Coassembly[b]

[a]MAG taxonomy was determined using the Genome Taxonomy Database (GTDB), which might be different than its taxonomy according to NCBI.

[b]Co-assemblies were performed with reads from all nine metagenomes sequenced, corresponding to Biosample numbers SAMN38883632, SAMN38883633, SAMN38883634, SAMN38883635, SAMN38883636, SAMN38883637, SAMN38883638, SAMN38883639, SAMN38883640. Sample names corresponding to each Biosample number are found in Table S9.

Section S6. Metatranscriptomics analyses

The TPM of a gene was calculated as follows:

$$TPM_i = \frac{q_i/l_i}{\sum_j(\, q_j/l_j)} \times 10^6$$

Where q_i is the sum of estimated count of *i*th gene, l_i is the effective length of *i*th gene. The values q_i and l_i were obtained from kallisto outputs. $\sum_j(q_j/l_j)$ corresponds to the sum of q_i/l_i for each gene.

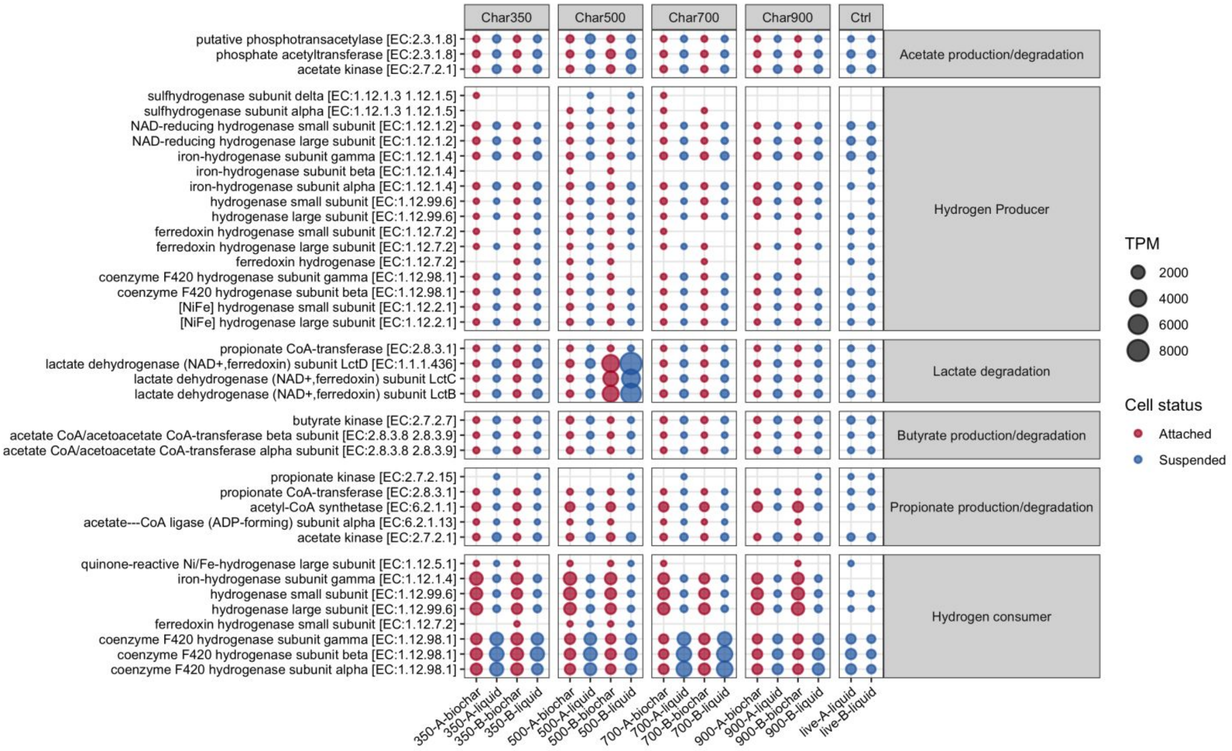

Figure S16. Expression levels (TPM) for genes encoding enzymes involved in acetate production/degradation, butyrate production (Acetate CoA-transferase and Butyryl-CoA:acetate CoA-transferase) lactate degradation (L-lactate dehydrogenase [EC:1.1.1.27], [EC:1.1.2.3], [EC:1.1.1.436], and propionate CoA-transferase [EC:2.8.3.1]), acetate formation (phosphate acetyltransferase [EC:2.3.1.8] and acetate kinase [EC:2.7.2.1]), propionate formation ((acetate kinase [EC:2.7.2.1], propionate kinase [EC:2.7.2.15], propionate CoA-transferase [EC:2.8.3.1], acetate---CoA ligase (ADP-forming) subunit alpha [EC:6.2.1.13], acetyl-CoA synthetase [EC:6.2.1.1], and propionyl-CoA synthetase [EC:6.2.1.17]), butyrate formation (butyrate kinase [EC:2.7.2.7], medium-chain acyl-CoA ligase / lipoate-activating enzyme [EC:6.2.1.2] and acetate CoA/acetoacetate CoA-transferase alpha subunit [EC:2.8.3.8]), and hydrogen consumption (i.e., hydrogenases from Dhc, *Desulfitobacterium* and methanogens) and hydrogen producer (hydrogenase gene expression from other microbes in SDC-9), from the SDC-9 microbial community in both biochar-attached and suspended cells in all treatments (Char350, Char500, Char700, and Char900) and the no material control (ctrl). The Enzyme Commission (EC) numbers provided indicate the enzyme entry in the KEGG database (https://www.genome.jp/), and includes relevant information about the biochemical pathways the enzyme participates in.

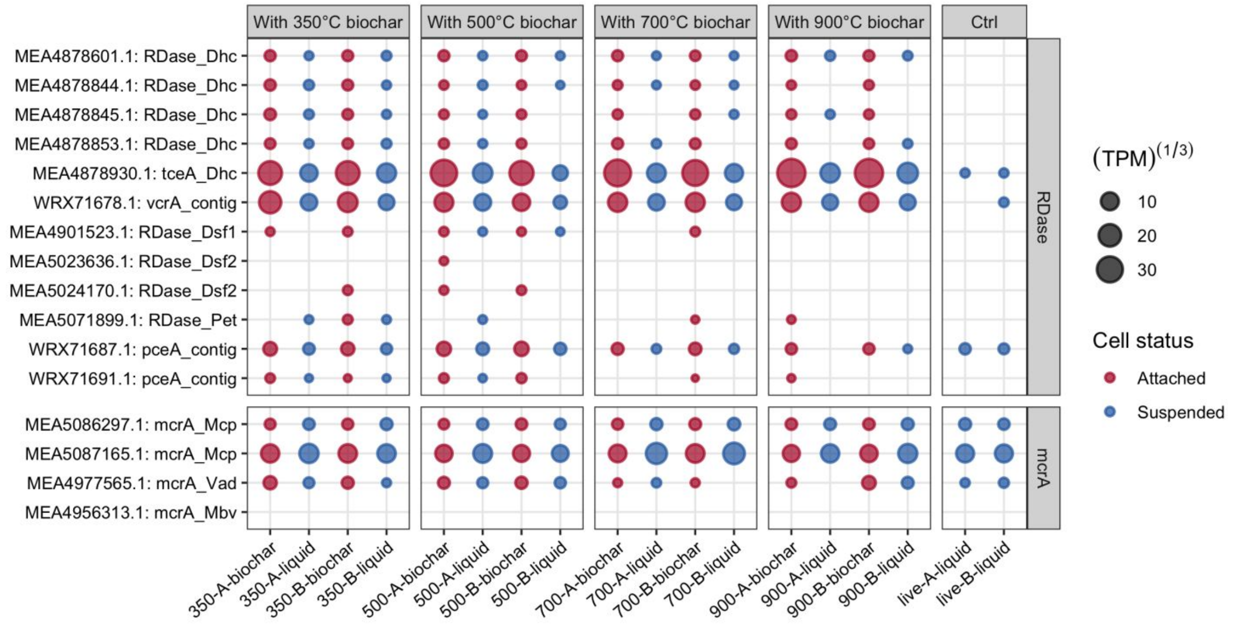

Figure S17. The expression of all RDase genes and *mcrA* (shown as $(\mathrm{TPM})^{1/3}$ to normalize the bubble size) across all SDC-9 metatranscriptomic samples, including the biochar treatments and the no material control (ctrl). Each gene is identified by its Genbank accession number (e.g. MEA4878601.1) with a suffix that indicates the taxonomy of the MAG harboring the gene. For example, the suffix "_Dhc" indicates the gene was found in the the *Dehalococcoides mccartyi* MAG. "_Dsf1" and "_Dsf2" are two different *Desulfitobacterium* MAGs, _Pet: *Petrimonas* MAG. RDase genes that reside on unbinned contigs (vcrA_contig; *Dsf-pceA*_contig) are also shown. The taxonomy of *mcrA* from methanogen MAGs is "_Mcp": *Methanocorpusculum*, "_Vad": Genus *VadinCA11*, and "_Mbv": *Methanobrevibacter*.

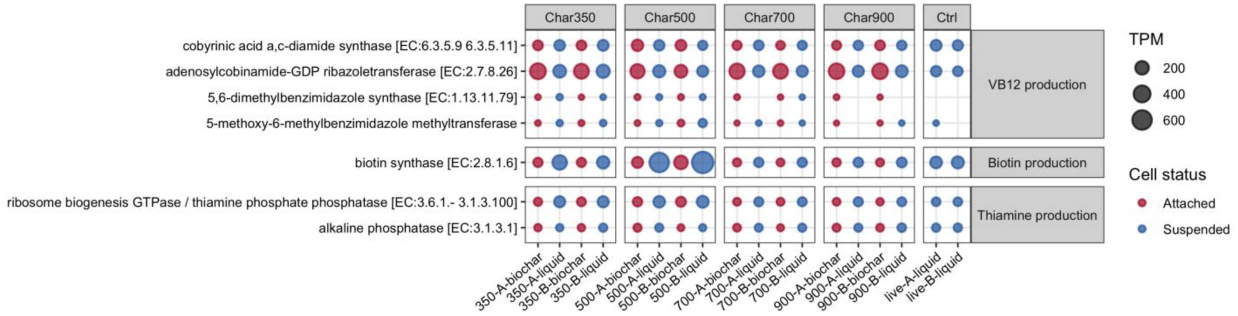

Figure S18. The expression of genes related to vitamin biosynthesis (i.e., B12, biotin, and thiamine) from other microorganisms in SDC-9 (excluding *Dehalococcoides*). Key enzymes in vitamin B12 biosynthesis include cobyrinic acid a,c-diamide synthase CbiA [EC:6.3.5.11] and adenosylcobinamide-GDP ribazoletransferase, CobS [EC:2.7.8.26] as shown in the Porphyrin metabolism KEGG pathway (map00860). The 5,6-dimethylbenzimidazole (DMB) synthase [EC:1.13.11.79] synthesizes the lower ligand of vitamin B12 (DMB). The 5-methoxy-6-methylbenzimidozole methyltransferase (BzaE) attaches the lower ligand (DMB) to the cobamide. Biotin synthase [EC:2.8.1.6] is the key enzyme involved in biotin synthesis. Key enzymes involved in thiamine biosynthesis include ribosome biogenesis GTPase [EC:3.1.3.100] and alkaline phosphatase [EC:3.1.3.1] as shown in the Thiamine metabolism KEGG pathway.

Section S7. Correlation analyses

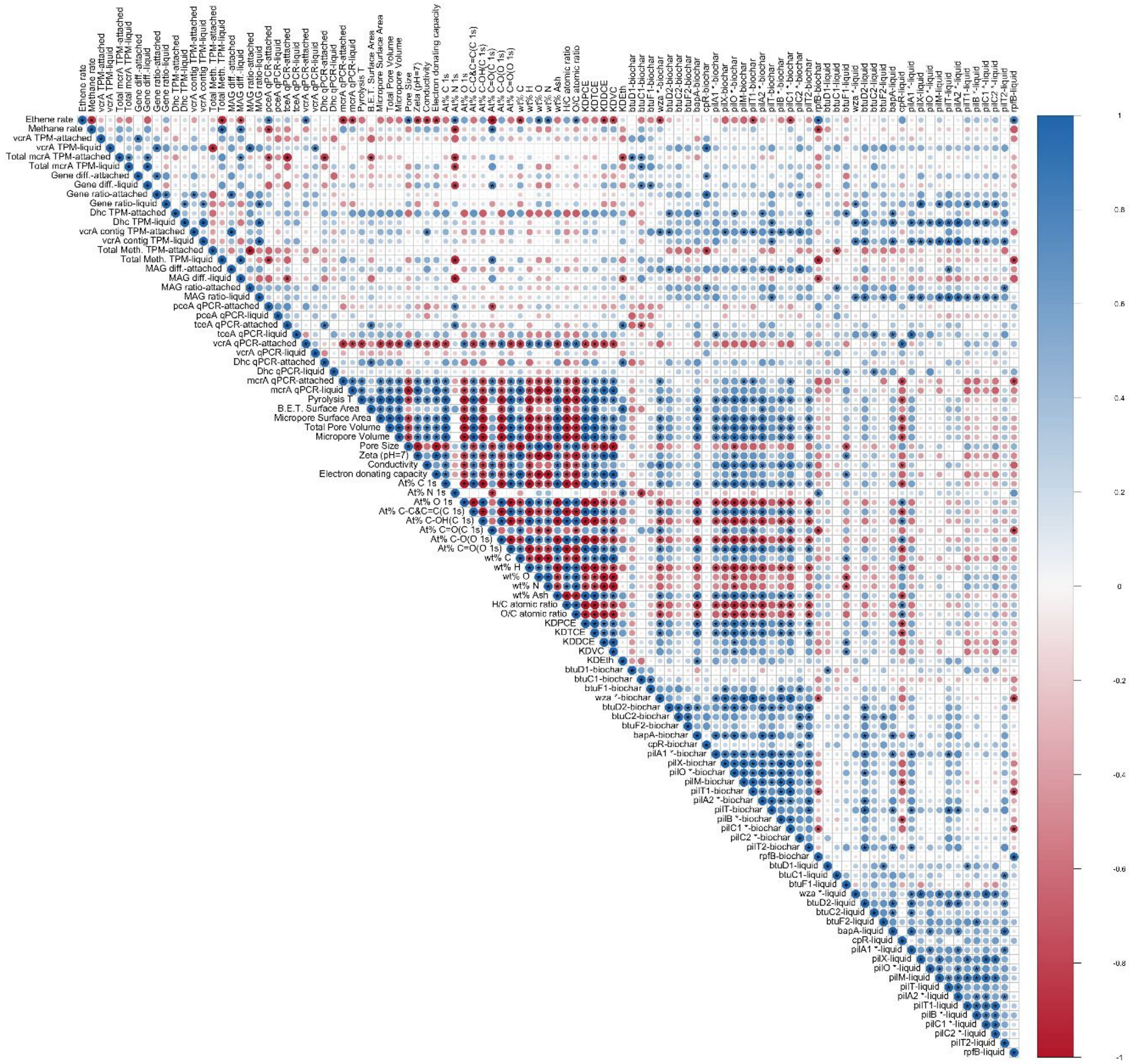

Figure S19. Spearman's ranking correlation matrix among ethene/methane production rate, the related biological information, material properties and growth-related gene expression from *Dehalococcoides*. Significant correlations were marked with "*". The subset of variables significantly correlated with ethene production are shown in Figure 4.

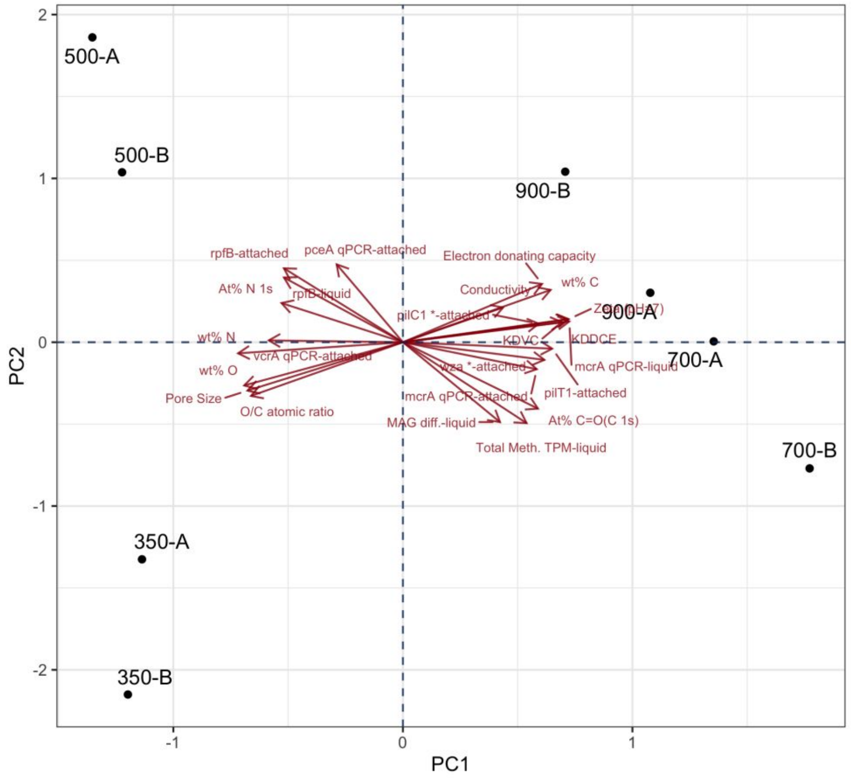

Figure S20. A PCA analysis biplot showing the sample distribution based on the variables significantly correlated with ethene production rate.

References

(1) Li, Z.; Mao, J.; Chu, W.; Xu, W., Probing the Surface Reactivity of Pyrogenic Carbonaceous Material (PCM) through Synthesis of PCM-Like Conjugated Microporous Polymers. *Environmental Science & Technology* **2019,** *53*, (13), 7673-7682.

(2) Dong, Q.; LeFevre, G. H.; Mattes, T. E., Black Carbon Impacts on Paraburkholderia xenovorans Strain LB400 Cell Enrichment and Activity: Implications toward Lower-Chlorinated Polychlorinated Biphenyls Biodegradation Potential. *Environ Sci Technol* **2024,** *58*, (8), 3895-3907.

(3) Yoshida, N.; Takahashi, N.; Hiraishi, A., Phylogenetic characterization of a polychlorinated-dioxin- dechlorinating microbial community by use of microcosm studies. *Appl Environ Microbiol* **2005,** *71*, (8), 4325-34.

(4) Ritalahti Kirsti, M.; Amos Benjamin, K.; Sung, Y.; Wu, Q.; Koenigsberg Stephen, S.; Löffler Frank, E., Quantitative PCR Targeting 16S rRNA and Reductive Dehalogenase Genes Simultaneously Monitors Multiple Dehalococcoides Strains. *Applied and Environmental Microbiology* **2006,** *72*, (4), 2765-2774.

(5) Fung, J. M.; Morris, R. M.; Adrian, L.; Zinder, S. H., Expression of reductive dehalogenase genes in Dehalococcoides ethenogenes strain 195 growing on tetrachloroethene, trichloroethene, or 2,3-dichlorophenol. *Appl Environ Microbiol* **2007,** *73*, (14), 4439-45.

(6) Cisek, A. A.; Bąk, I.; Cukrowska, B., Improved Quantitative Real-Time PCR Protocol for Detection and Quantification of Methanogenic Archaea in Stool Samples. *Microorganisms* **2023,** *11*, (3), 660.

(7) Michalsen, M. M.; Kara Murdoch, F.; Löffler, F. E.; Wilson, J.; Hatzinger, P. B.; Istok, J. D.; Mullins, L.; Hill, A.; Murdoch, R. W.; Condee, C.; Kucharzyk, K. H., Quantitative Proteomics and Quantitative PCR as Predictors of cis-1,2-Dichlorethene and Vinyl Chloride Reductive Dechlorination Rates in Bioaugmented Aquifer Microcosms. *ACS ES&T Engineering* **2022,** *2*, (1), 43-53.

(8) Bradley, P. M., History and Ecology of Chloroethene Biodegradation: A Review. *Bioremediation Journal* **2003,** *7*, (2), 81-109.

(9) Mattes, T. E.; Alexander, A. K.; Coleman, N. V., Aerobic biodegradation of the chloroethenes: pathways, enzymes, ecology, and evolution. *FEMS Microbiol Rev* **2010,** *34*, (4), 445-75.

(10) Magnuson, J. K.; Romine, M. F.; Burris, D. R.; Kingsley, M. T., Trichloroethene reductive dehalogenase from Dehalococcoides ethenogenes: sequence of tceA and substrate range characterization. *Applied and environmental microbiology* **2000,** *66*, (12), 5141-5147.

(11) Müller, J. A.; Rosner, B. M.; Von Abendroth, G.; Meshulam-Simon, G.; McCarty, P. L.; Spormann, A. M., Molecular identification of the catabolic vinyl chloride reductase from Dehalococcoides sp. strain VS and its environmental distribution. *Applied and environmental microbiology* **2004,** *70*, (8), 4880-4888.

(12) Parthasarathy, A.; Stich, T. A.; Lohner, S. T.; Lesnefsky, A.; Britt, R. D.; Spormann, A. M., Biochemical and EPR-spectroscopic investigation into heterologously expressed vinyl chloride reductive dehalogenase (VcrA) from Dehalococcoides mccartyi strain VS. *J Am Chem Soc* **2015,** *137*, (10), 3525-3532.

(13) Fathepure, B. Z.; Boyd, S. A., Dependence of tetrachloroethylene dechlorination on methanogenic substrate consumption by Methanosarcina sp. strain DCM. *Appl Environ Microbiol* **1988,** *54*, (12), 2976-80.

(14) Bolger, A. M.; Lohse, M.; Usadel, B., Trimmomatic: a flexible trimmer for Illumina sequence data. *Bioinformatics* **2014,** *30*, (15), 2114-2120.

(15) Li, D.; Liu, C.-M.; Luo, R.; Sadakane, K.; Lam, T.-W., MEGAHIT: an ultra-fast single-node solution for large and complex metagenomics assembly via succinct de Bruijn graph. *Bioinformatics* **2015,** *31*, (10), 1674-1676.

(16) Seemann, T., Prokka: rapid prokaryotic genome annotation. *Bioinformatics* **2014,** *30*, (14), 2068-2069.
(17) Langmead, B.; Salzberg, S. L., Fast gapped-read alignment with Bowtie 2. *Nature Methods* **2012,** *9*, (4), 357-359.
(18) Kang, D.; Li, F.; Kirton, E. S.; Thomas, A.; Egan, R. S.; An, H.; Wang, Z., MetaBAT 2: an adaptive binning algorithm for robust and efficient genome reconstruction from metagenome assemblies. *PeerJ Preprints* **2019,** *7*, e27522v1.
(19) Parks, D. H.; Imelfort, M.; Skennerton, C. T.; Hugenholtz, P.; Tyson, G. W., CheckM: assessing the quality of microbial genomes recovered from isolates, single cells, and metagenomes. *Genome Res* **2015,** *25*, (7), 1043-1055.
(20) Eren, A. M.; Kiefl, E.; Shaiber, A.; Veseli, I.; Miller, S. E.; Schechter, M. S.; Fink, I.; Pan, J. N.; Yousef, M.; Fogarty, E. C.; Trigodet, F.; Watson, A. R.; Esen, Ö. C.; Moore, R. M.; Clayssen, Q.; Lee, M. D.; Kivenson, V.; Graham, E. D.; Merrill, B. D.; Karkman, A.; Blankenberg, D.; Eppley, J. M.; Sjödin, A.; Scott, J. J.; Vázquez-Campos, X.; McKay, L. J.; McDaniel, E. A.; Stevens, S. L. R.; Anderson, R. E.; Fuessel, J.; Fernandez-Guerra, A.; Maignien, L.; Delmont, T. O.; Willis, A. D., Community-led, integrated, reproducible multi-omics with anvi'o. *Nature Microbiology* **2021,** *6*, (1), 3-6.
(21) Olm, M. R.; Brown, C. T.; Brooks, B.; Banfield, J. F., dRep: a tool for fast and accurate genomic comparisons that enables improved genome recovery from metagenomes through de-replication. *The ISME Journal* **2017,** *11*, (12), 2864-2868.
(22) Chaumeil, P.-A.; Mussig, A. J.; Hugenholtz, P.; Parks, D. H., GTDB-Tk: a toolkit to classify genomes with the Genome Taxonomy Database. *Bioinformatics* **2020,** *36*, (6), 1925-1927.
(23) Hyatt, D.; Chen, G.-L.; LoCascio, P. F.; Land, M. L.; Larimer, F. W.; Hauser, L. J., Prodigal: prokaryotic gene recognition and translation initiation site identification. *BMC Bioinformatics* **2010,** *11*, (1), 119.
(24) Eddy, S. R., Accelerated Profile HMM Searches. *PLOS Computational Biology* **2011,** *7*, (10), e1002195.
(25) Matsen, F. A.; Kodner, R. B.; Armbrust, E. V., pplacer: linear time maximum-likelihood and Bayesian phylogenetic placement of sequences onto a fixed reference tree. *BMC Bioinformatics* **2010,** *11*, (1), 538.
(26) Jain, C.; Rodriguez-R, L. M.; Phillippy, A. M.; Konstantinidis, K. T.; Aluru, S., High throughput ANI analysis of 90K prokaryotic genomes reveals clear species boundaries. *Nature Communications* **2018,** *9*, (1), 5114.
(27) Price, M. N.; Dehal, P. S.; Arkin, A. P., FastTree 2 – Approximately Maximum-Likelihood Trees for Large Alignments. *PLOS ONE* **2010,** *5*, (3), e9490.
(28) Tatusova, T.; DiCuccio, M.; Badretdin, A.; Chetvernin, V.; Nawrocki, E. P.; Zaslavsky, L.; Lomsadze, A.; Pruitt, K. D.; Borodovsky, M.; Ostell, J., NCBI prokaryotic genome annotation pipeline. *Nucleic Acids Research* **2016,** *44*, (14), 6614-6624.
(29) Aramaki, T.; Blanc-Mathieu, R.; Endo, H.; Ohkubo, K.; Kanehisa, M.; Goto, S.; Ogata, H., KofamKOALA: KEGG Ortholog assignment based on profile HMM and adaptive score threshold. *Bioinformatics* **2020,** *36*, (7), 2251-2252.
(30) Mistry, J.; Chuguransky, S.; Williams, L.; Qureshi, M.; Salazar, Gustavo A.; Sonnhammer, E. L. L.; Tosatto, S. C. E.; Paladin, L.; Raj, S.; Richardson, L. J.; Finn, R. D.; Bateman, A., Pfam: The protein families database in 2021. *Nucleic Acids Research* **2021,** *49*, (D1), D412-D419.
